# Strategies for Enhancing BiVO_4_ Photoanodes for PEC Water Splitting: A State-of-the-Art Review

**DOI:** 10.3390/nano15191494

**Published:** 2025-09-30

**Authors:** Binh Duc Nguyen, In-Hee Choi, Jae-Yup Kim

**Affiliations:** Department of Chemical Engineering, Konkuk University, Seoul 05029, Republic of Korea; binhjonny22@konkuk.ac.kr (B.D.N.); choiih1303@konkuk.ac.kr (I.-H.C.)

**Keywords:** bismuth vanadate (BiVO_4_), photoelectrochemical (PEC) water splitting, synthesis method, photocatalysis

## Abstract

Bismuth vanadate (BiVO_4_) has attracted significant attention as a photoanode material for photoelectrochemical (PEC) water splitting due to its suitable bandgap (~2.4 eV), strong visible light absorption, chemical stability, and cost-effectiveness. Despite these advantages, its practical application remains constrained by intrinsic limitations, including poor charge carrier mobility, short diffusion length, and sluggish oxygen evolution reaction (*OER*) kinetics. This review critically summarizes recent advancements aimed at enhancing BiVO_4_ PEC performance, encompassing synthesis strategies, defect engineering, heterojunction formation, cocatalyst integration, light-harvesting optimization, and stability improvements. Key fabrication methods—such as solution-based, vapor-phase, and electrochemical approaches—along with targeted modifications, including metal/nonmetal doping, surface passivation, and incorporation of electron transport layers, are discussed. Emphasis is placed on strategies to improve light absorption, charge separation efficiency (*η_sep_*), and charge transfer efficiency (*η_trans_*) through bandgap engineering, optical structure design, and catalytic interface optimization. Approaches to enhance stability via protective overlayers and electrolyte tuning are also reviewed, alongside emerging applications of BiVO_4_ in tandem PEC systems and selective solar-driven production of value-added chemicals, such as *H*_2_*O*_2_. Finally, critical challenges, including the scale-up of electrode fabrication and the elucidation of fundamental reaction mechanisms, are highlighted, providing perspectives for bridging the gap between laboratory performance and practical implementation.

## 1. Introduction

The escalating concerns over global warming and the depletion of fossil fuels have intensified the demand for clean and renewable energy sources [1,2,3,4]. In this context, hydrogen has emerged as a promising energy carrier due to its high energy density and environmentally benign characteristics [5,6,7,8]. In particular, photoelectrochemical (PEC) water splitting utilizing solar energy has attracted considerable interest as a sustainable approach for hydrogen production. PEC water splitting provides an efficient and cost-effective route to directly convert solar energy into chemical energy, exhibiting higher energy conversion efficiency compared to conventional electrolysis methods [9,10,11].

In PEC systems, the selection of photoelectrode materials is critical for achieving high efficiency and stability. An ideal photoelectrode should possess strong visible light absorption, efficient charge separation and transport, appropriate band alignment, and chemical stability in aqueous environments [4,12,13,14]. Among various semiconductors, bismuth vanadate (BiVO_4_) has drawn substantial attention as a material that meets these requirements [15,16,17].

BiVO_4_ exhibits several advantageous properties for PEC hydrogen production, including a suitable bandgap (~2.4 eV), strong visible light absorption, cost-effectiveness, and environmental benignity [18,19,20,21]. Its band structure is particularly favorable for water oxidation, making BiVO_4_ a widely studied n-type photoelectrode [22,23,24]. Moreover, BiVO_4_ demonstrates high chemical stability, resulting in minimal performance degradation under long-term operation [25,26,27].

Despite these promising characteristics, BiVO_4_ faces intrinsic limitations that hinder its practical application. Low charge carrier mobility, short minority carrier diffusion length, and sluggish oxygen evolution reaction (*OER*) kinetics are major challenges [28,29,30], leading to a notable gap between theoretical and actual PEC performance. To overcome these limitations, various strategies have been explored, including nanostructure engineering, heteroatom doping, heterojunction formation, cocatalyst integration, and surface passivation [31,32,33].

Nanostructure engineering increases the surface area and shortens charge transfer distances, thereby enhancing the photocurrent [34,35]. Heteroatom doping improves electrical conductivity and tunes the band structure [36,37]. Heterojunction formation promotes charge separation and extends light absorption [38,39]. Cocatalyst integration lowers the overpotential for *OER* and accelerates reaction rates [40,41], while surface passivation mitigates surface defects and suppresses charge recombination [42,43]. These strategies are often combined to achieve significant improvements in BiVO_4_ PEC performance.

This review aims to provide a comprehensive overview of recent research trends in BiVO_4_-based PEC hydrogen production. We discussed the fundamental properties of BiVO_4_, synthesis methods, performance enhancement strategies, and PEC system configurations.

## 2. Principle of Operation for Photoanode in PEC Water Splitting

Electrochemical (EC) water splitting is initiated by photon absorption in the semiconductor material of the photoelectrode. For this process to occur, incident photons must possess sufficient energy to excite electrons from the valence band (*VB*) to the conduction band (*CB*), i.e., when the photon energy (*hν*) is equal to or greater than the semiconductor’s bandgap energy (*E_g_*), generating electron–hole pairs that drive subsequent chemical reactions:

(1) $$SC+hv\left(\geq E_{g}\right)\to e_{CB}^{-}+h_{VB}^{+}:Light\;absorption$$

The photogenerated charge carriers participate in spatially separated redox reactions. Electrons in the *CB* (*e_CB_^−^*) with potentials more negative than 0.0 *V_RHE_* migrate to the photoelectrode surface and reduce protons to hydrogen, typically facilitated by a hydrogen evolution cocatalyst (HEC):

(2) $$4H^{+}+4e_{CB}^{-}\to 2H_{2}\;:HER$$

Simultaneously, holes in the *VB* (*h_VB_^+^*) with potentials more positive than 1.23 *V_RHE_* can oxidize water to oxygen, typically facilitated by an oxygen evolution cocatalyst (OEC):

(3) $$2H_{2}O+4h_{VB}^{+}\to O_{2}+4H^{+}:OER$$

Overall, water splitting proceeds as

(4) $$2H_{2}O\to 2H_{2}+O_{2},\;∆G^{{}^{\circ}}=238\;kJ\;{mol}^{-1}$$

To theoretically split water, a semiconductor must satisfy two criteria: (i) a bandgap energy (*E_g_*) exceeding 1.23 eV, and (ii) a *CB* edge more negative than 0.0 *V_RHE_* and a *VB* edge more positive than 1.23 *V_RHE_* [44]. In practice, overpotentials associated with the hydrogen and oxygen evolution reactions—approximately 0.05 V for HER and 0.25 V for *OER*—require a larger effective bandgap (>1.5 eV) to drive the reactions efficiently.

Hydrogen production efficiency is often limited by electron–hole recombination, which reduces the availability of charge carriers for water splitting reactions. PEC cells employ a dual-electrode configuration, producing *O*_2_ at the anode and *H*_2_ at the cathode [45]. This spatial separation of oxidation and reduction reactions enhances energy conversion efficiency, improves operational stability, and reduces recombination losses compared to conventional photocatalytic systems using dispersed semiconductor powders. By maintaining distinct reaction sites, PEC cells optimize light absorption and charge carrier dynamics, enabling more reliable hydrogen production.

**Figure 1:**
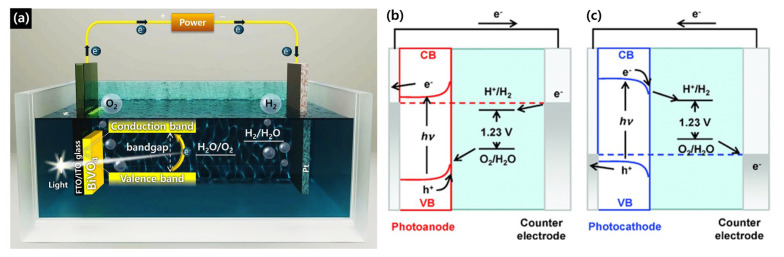
(**a**) Schematic of PEC cell in two-electrode configuration. Graphic visualization of PEC energy diagrams for PEC water splitting using (**b**) a photoanode; (**c**) a photocathode. Reprinted with permission from Ref. [46], Copyright © 2022, ECS Advances.

Figure 1 illustrates that when the semiconductor absorbs light, electron–hole pairs are generated in the *CB* and *VB*, respectively. Due to the band bending at the semiconductor–electrolyte interface, holes migrate toward the surface, where they initiate the *OER*, oxidizing water. Concurrently, the photogenerated electrons are transported through a transparent conducting substrate, such as fluorine-doped tin oxide (FTO), and collected into an external circuit. An applied potential (*E_app_*) is often used to provide the necessary energetic boost for the electrons, enabling them to travel to the metallic cathode. At the cathode, these electrons drive the hydrogen evolution reaction (HER), leading to hydrogen production. This synchronized process of water oxidation and reduction effectively converts light energy into chemical energy.

This bias voltage is generally supplied by a photovoltaic (PV) cell positioned behind the photoanode, which captures any transmitted photons and converts them into electrical power. In this tandem configuration, the PV device enhances the overall efficiency of the PEC system. The primary goal of this design is to achieve effective and sustainable hydrogen generation via water splitting.

To better evaluate the efficiency and intrinsic limitations of charge transfer in photoelectrodes, a well-established experimental methodology allows for the quantification of key performance parameters, including the photocurrent density (*J_ph_*), absorbed photon flux (*J_abs_*), and the rates of surface recombination (*J_sr_*) and bulk recombination (*J_br_*) [17]. Photogenerated charge carriers on a photoanode face two competing fates: they can either contribute to the desired photocurrent or undergo recombination, which constitutes a major energy loss pathway. Surface recombination occurs when charges recombine at the interface before reacting with the electrolyte, whereas bulk recombination refers to the loss of carriers within the semiconductor material. The balance between these two pathways is a critical factor determining the overall performance and efficiency of the photoanode in solar-driven water splitting.

When highly reactive species, such as hydrogen peroxide (*H*_2_*O*_2_) or sodium sulfite (Na_2_SO_3_), are introduced as sacrificial reagents, the surface recombination rate is significantly mitigated. Due to their strong tendency to undergo oxidation, these species provide kinetically favorable pathways for hole transfer to the electrolyte. Under such conditions, photogenerated holes are efficiently consumed in oxidation reactions, preventing their recombination with electrons at the photoanode surface.

By analyzing these recombination pathways, researchers can gain insights into the key factors limiting charge carrier dynamics, which in turn determine the overall efficiency of a photoelectrode. During the water oxidation reaction, the net photocurrent—denoted as *J_H_*_2*O*_—reflects the portion of absorbed photon flux (*J_abs_*) that successfully contributes to oxygen evolution after accounting for losses due to surface and bulk recombination:

(5) $$J_{H_{2}O}=J_{abs}-(J_{sr}+J_{br})$$

The establishment of a semiconductor–electrolyte junction, commonly referred to as a semiconductor–liquid (SCL) junction, is a crucial process in PEC cells featuring photoelectrodes without a buried junction. The SCL junction facilitates the separation of photogenerated electron–hole pairs at the interface, directing electrons toward the external circuit and holes toward the electrolyte. Unlike buried junctions formed by stacking two semiconductors, the SCL junction operates directly at the semiconductor–electrolyte boundary, presenting unique challenges and opportunities for optimizing PEC efficiency and stability.

## 3. Synthesis Methods of BiVO_4_ Photoelectrodes

The synthesis method plays a critical role in achieving high-efficiency photoelectrodes [16], as it directly determines the nature and concentration of defects within the material. These defects, in turn, influence the overall conductivity, its type, and the effectiveness of any applied modifications. Table 1 provides a summary of common synthetic techniques and the resulting morphologies of BiVO_4_. The fabrication of BiVO_4_ on substrates—primarily fluorine-doped tin oxide (FTO)—typically begins with the deposition of bismuth (Bi) and vanadium (V) precursors, which are subsequently converted in situ into a BiVO_4_ film at temperatures ranging from 400 to 500 °C, either in air or under a controlled atmosphere.

Most BiVO_4_ films produced by this method exhibit a monoclinic phase with relatively poor crystallinity, though some show characteristics of a tetragonal phase, as indicated by the weak splitting of the XRD peak around 2θ ≈ 19° [55]. Despite the low crystallinity, BiVO_4_ possesses “defect tolerance” [2], which allows it to maintain efficient charge separation. As a result, crystallinity is not the primary factor determining the performance of BiVO_4_ in PEC applications.

**Figure 2:**
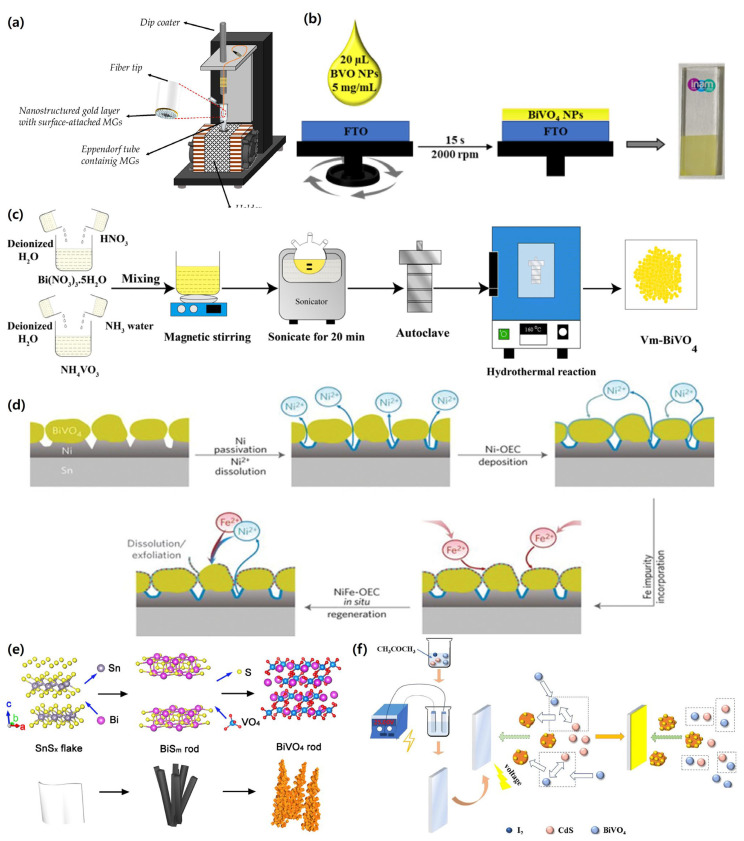
Representative morphologies for selected BiVO_4_ synthesis routes. (**a**) Automatic dip coating machine. Reprinted with permission from ref. [48], Copyright © MDPI, 2018. Schematic representation for the synthesis by (**b**) hydrothermal, reprinted with permission from ref. [63], Copyright © Royal Society of Chemistry, 2023; (**c**) spin coating, reprinted with permission from ref. [64], Copyright © MDPI, 2021; (**d**) mechanism of the in situ generation of NiFeO*_x_* catalyst on BiVO_4_, reprinted with permission from ref. [65], Copyright © 2020, Wiley-VCH GmbH; (**e**) ion exchange technique, reprinted with permission from ref. [66], Copyright © 2016, American Chemical Society; (**f**) electrophoretic deposition of BiVO_4_, reprinted with permission from ref. [67], Copyright © 2021, Research square.

Figure 2 presents representative exemplars for selected synthesis routes discussed in Section 3.1,Section 3.2,Section 3.3,Section 3.4,Section 3.5; methods that are not depicted in Figure 2 are referenced textually 

Mapping of panels to sections: Figure 2a→Section 3.1 (Hydrothermal), Figure 2b→Section 3.2 (Electrodeposition), Figure 2c→Section 3.3 (BiOI conversion), Figure 2d→Section 3.4 (Sol–gel). Methods in Section 3.5 (e.g., aerosol/ALD) are illustrated in Figure 2e,f.

### 3.1. Preparation of Nanoporous BiVO_4_ Films

A representative hydrothermal morphology (nanoporous BiVO_4_) is shown in Figure 2a, a variety of solution-based approaches—such as soaking, dip coating, and impregnation—have been extensively employed for the preparation of BiVO_4_ photoanodes [56,57,58,59]. In the soaking method, the substrate is immersed in a precursor solution for an extended duration, allowing surface chemical reactions to occur; although straightforward, this technique often requires long processing times [56]. Dip-coating provides greater control by immersing the substrate in the solution followed by withdrawal at a regulated speed, enabling tunable film thickness through adjustment of the withdrawal rate [57]. Impregnation involves introducing the precursor solution into porous supports, which are subsequently dried and calcined, making this approach particularly effective for producing catalyst-supported BiVO_4_ materials with high specific surface area [58,59].

### 3.2. Spin Coating, Drop-Casting, Spray Pyrolysis, and Electrospray Deposition

An electrodeposited BiOI precursor prior to conversion is illustrated in Figure 2; and thin-film deposition techniques—including spin coating, drop-casting, spray pyrolysis, and electrospray deposition (ESD)—have also been applied in BiVO_4_ fabrication [20,60,61,62]. In spin coating, a small quantity of precursor solution is dispensed onto a rotating substrate, where centrifugal forces ensure the formation of a uniform thin film [60]. Drop-casting involves placing droplets of the precursor solution onto the substrate and allowing them to dry under ambient conditions; however, this method can result in film thickness variations [20]. Spray pyrolysis directs an aerosolized precursor onto a heated substrate, initiating pyrolysis and forming a continuous thin film [61]. Electrospray deposition (ESD) employs a high-voltage electric field to generate a fine mist of the precursor solution, enabling precise deposition of nanoparticles or uniform films onto the substrate surface [62].

### 3.3. Sol–Gel Method, Wet-Chemical Process, Hydrothermal, Solvothermal, SILAR

Cross-sectional features after BiOI→BiVO_4_ conversion are shown in Figure 2c; enlarged views and phase verification are compiled; solution-based synthesis routes—including hydrothermal, solvothermal, wet-chemical, successive ionic layer adsorption and reaction (SILAR), and sol–gel processes—are widely utilized for fabricating nanostructured BiVO_4_ [68,69,70,71,72]. Hydrothermal synthesis, conducted in an aqueous medium under elevated temperature and pressure, enables the growth of highly crystalline BiVO_4_ [68]. Solvothermal synthesis, analogous to hydrothermal synthesis but carried out in organic solvents, provides additional versatility in tailoring particle size and morphology [69]. Wet-chemical methods encompass various liquid-phase synthesis techniques, offering flexibility in adjusting material composition and properties [70]. The SILAR technique, a layer-by-layer deposition process, is effective for thin-film growth under ambient conditions, making it a cost-efficient route for large-scale fabrication [71]. The sol–gel method involves generating a sol from metal precursors in solution, which subsequently transforms into a gel and undergoes heat treatment to yield solid BiVO_4_, facilitating uniform nanostructure formation at low temperatures and scalability [72].

### 3.4. Chemical Vapor Deposition (CVD), Reactive Co-Sputtering, and Atomic Layer Deposition (ALD)

A dense, sol–gel-derived BiVO_4_ layer is depicted in Figure 2d; roughness tuning vs. annealing temperature is summarized; post-synthesis modification techniques—such as annealing, plasma treatment, and surface grafting—are commonly employed to enhance the structural, electronic, and interfacial properties of BiVO_4_ thin films [73,74,75,76]. Annealing at elevated temperatures improves crystallinity, reduces structural defects, and enhances charge transport [73]. Plasma treatment introduces reactive species to the film surface, enabling controlled modification of surface chemistry and improving catalytic activity, wettability, or interfacial charge transfer [74,75]. Surface grafting involves covalently attaching functional molecules to the BiVO_4_ surface, allowing tailored chemical functionalities that enhance PEC performance, stability, or compatibility with cocatalysts [76].

### 3.5. In Situ Coordination, Ion Exchange Technique, and Grafting

As shown in Figure 2e,f, Chemical modification strategies—including in situ coordination, ion exchange, and surface grafting—allow precise tailoring of BiVO_4_ composition and structure during or after synthesis [77,78,79,80,81,82]. In situ coordination facilitates controlled formation of coordination complexes during synthesis, influencing crystal structure, morphology, and optoelectronic properties [77]. Ion exchange techniques substitute selected ions within the BiVO_4_ lattice, enabling precise doping or compositional tuning to enhance optical absorption and catalytic activity [78]. Grafting, as a post-synthesis surface functionalization, allows covalent attachment of molecular species or functional groups to the BiVO_4_ surface, improving surface reactivity, stability, or compatibility with cocatalysts [79].

Electrochemical fabrication methods—including electrodeposition and photoelectrophoretic deposition (PEPD)—have also been successfully applied for BiVO_4_ photoanodes [80,81,82]. Electrodeposition uses an externally applied current to deposit BiVO_4_ from a precursor solution onto a conductive substrate, providing precise control over film thickness, morphology, and stoichiometry by tuning deposition parameters [80,81]. PEPD employs a photoinduced electric field to drive nanoparticle deposition onto the substrate, forming uniform, size-controlled nanoparticle-based BiVO_4_ films with homogeneous distribution [82].

## 4. Key Factors Influencing the PEC Performance of the BIVO_4_ Photoanode

The photocurrent density (*J_PEC_*) is a fundamental parameter for assessing the PEC performance of a photoelectrode. For a BiVO_4_ photoanode, *J_PEC_* can be calculated according to the following equation [31]:

(6) $$J_{PEC}=J_{max}\times LHE\times \eta_{trans}\times \eta_{sep}$$

(7) $$Arrhenius\;equation:k=Aexp(-\frac{E_{AOP}}{RT})$$

In Equation (6), *J_ma__x_* represents the theoretical maximum photocurrent density attainable by BiVO_4_, which has been reported to be approximately 7.5 mA/cm^2^. According to this relationship, the PEC efficiency of BiVO_4_-based photoanodes can be significantly improved by enhancing the light-harvesting efficiency (*LHE*), charge separation efficiency *η_sep_*, and charge transport efficiency *η_trans_*). Optimization of one or more of these factors—either individually or synergistically—is crucial for maximizing the overall performance of the photoanode in solar water splitting applications [83].

The rapid advancement of BiVO_4_-based photoanodes has been driven by various modification strategies, including elemental doping, post-treatment methods, incorporation of oxygen evolution catalysts (OECs), and integration of electron transport layers (ETLs) or hole transport layers (HTLs), in addition to nanostructuring techniques, as summarized in Table 2. The effectiveness of these strategies is multifaceted; for instance, ETLs not only reduce charge recombination at the interface between BiVO_4_ and ETL, as well as within the bulk of the photoelectrode, but they also significantly enhance surface charge transfer. This improvement arises from the influence of the underlying ETL on the deposition and structural characteristics of the overlying BiVO_4_ layer.

**Figure 3:**
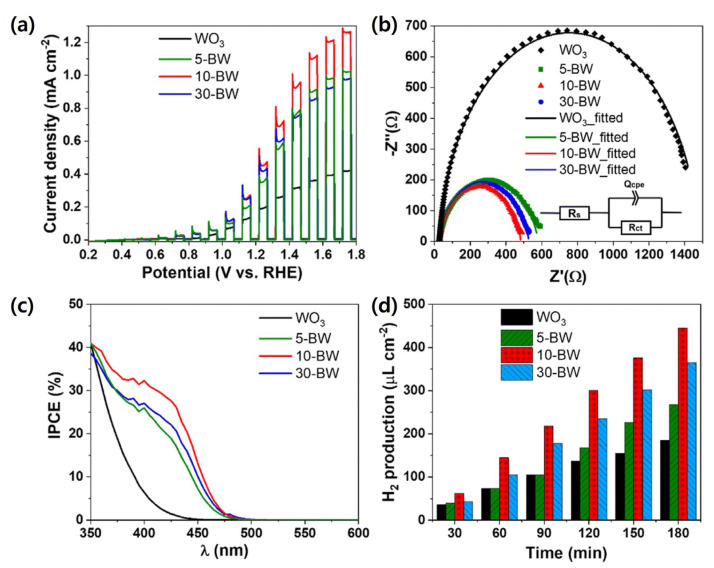
PEC performances of the WO_3_ nanocorals and BiVO_4_/WO_3_ (BW) photoanodes of 5-BW, 10-BW, and 30-BW: (**a**) photocurrent densities by linear sweep voltammetry (LSV), (**b**) Nyquist plots and equivalent circuits, (**c**) PEC *H*_2_ production diagram as a function of time, and (**d**) IPCE spectra. Reprinted with permission from Ref. [106], Copyright © 2024, Royal Society of Chemistry.

As shown in Figure 3, Park et al. developed a heterostructured photoanode by decorating anodic WO_3_ “nanocoral” arrays with ultrafine BiVO_4_ nanoparticles (~10 nm) via spin coating. This linked configuration established an efficient electron transfer pathway while preserving the nanocoral morphology. Under simulated solar illumination, the BiVO_4_/WO_3_ nanocoral electrode exhibited a photocurrent density of approximately 2.4 times that of bare WO_3_. More notably, incident photon-to-current conversion efficiency (IPCE) at 410 nm increased by a factor of 8.3, indicating substantially enhanced light absorption and charge carrier utilization. This study demonstrates that nanoscale heterojunction engineering combined with optimized morphology control can synergistically improve both optical harvesting and interfacial charge dynamics in PEC systems [106].

### 4.1. Introducing Extrinsic/Intrinsic Defects Through Doping

Doping plays a critical role in enhancing the performance of BiVO_4_ photoelectrodes by modulating their electronic and chemical properties. A key strategy for improving PEC efficiency involves either the introduction of intrinsic defects, such as oxygen vacancies, or the incorporation of foreign metal ions into the crystal lattice. Recent studies have provided valuable insights into different approaches for defect engineering, highlighting how such modifications can influence charge separation and transport processes.

**Figure 4:**
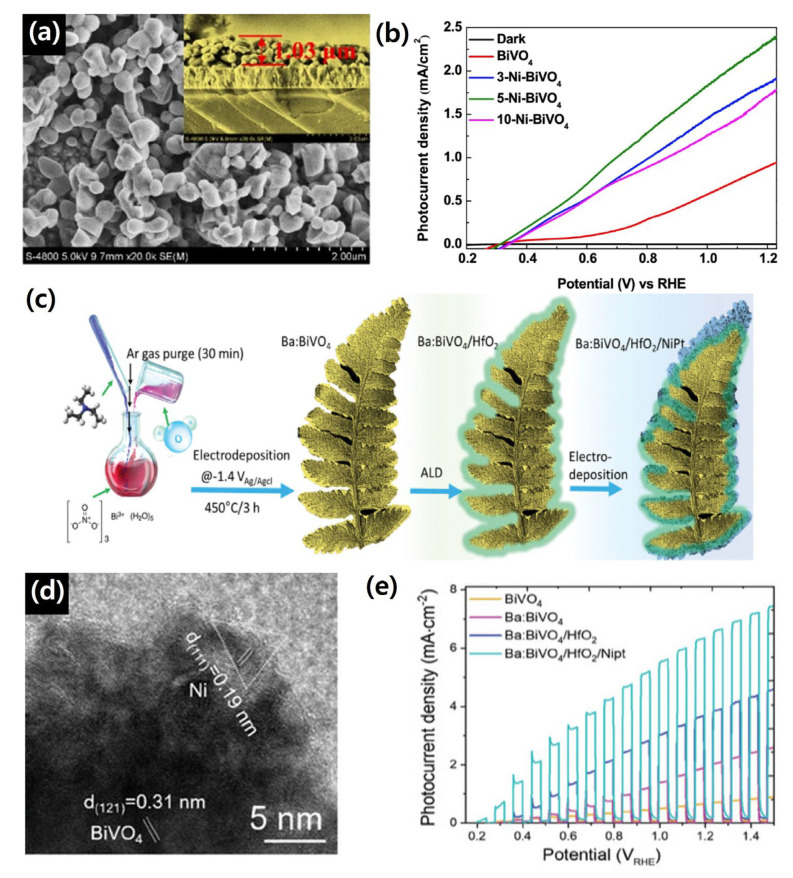
(**a**) SEM images of Ni-doped BiVO_4_. (**b**) *J*–*V* curves of pure BiVO_4_ and Ni-doped BiVO_4_. Reprinted with permission from Ref. [107], Copyright © 2024, Springer Nature. (**c**) Schematic illustration of photoanode fabrication process (**d**) HR-TEM images of Ba:BiVO_4_/HfO_2_/NiPt. (**e**) *J*–*V* curves of pure BiVO_4_ and Ni-doped BiVO_4_. Reprinted with permission from Ref. [108], Copyright © 2024, Wiley-VCH GmbH.

Kong et al. demonstrated that Ni doping in BiVO_4_, achieved via electrodeposition, significantly enhances PEC performance through defect engineering. Ni-doped BiVO_4_ samples were categorized according to Ni content. The nanoparticle size decreased with increasing Ni content: 500 nm for pristine BiVO_4_, 440 nm for 3-Ni-BiVO_4_, 310 nm for 5-Ni-BiVO_4_, and 200 nm for 10-Ni-BiVO_4_. Controlled Ni incorporation promotes the formation of oxygen vacancies and surface V^4+^ species, which simultaneously improve charge separation and catalytic activity at the electrode–electrolyte interface. These synergistic effects effectively suppress recombination and accelerate charge transport. The morphology of Ni-doped BiVO_4_ are shown in Figure 4a [107]. As a result, the optimized 5-Ni-BiVO_4_ exhibited a photocurrent density of 2.39 mA/cm^2^ at 1.23 *V_RHE_*, approximately 2.5 times higher than pristine BiVO_4_ (0.94 mA/cm^2^), along with an IPCE of 45% (400–450 nm) and an ABPE of 0.55%, as shown is Figure 4b. In contrast, excessive Ni in 10-Ni-BiVO_4_ introduced additional recombination centers, highlighting the importance of finely tuning oxygen vacancy concentrations to achieve optimal PEC efficiency [107].

Shin et al. employed a synergistic combination of Ba doping, HfO_2_ passivation, and NiPt alloy cocatalyst decoration to enhance charge transport and interfacial kinetics. Ba doping into the BiVO_4_ lattice introduced beneficial defect states, improving carrier separation. A thin HfO_2_ layer, deposited via atomic layer deposition (ALD), served as a surface passivation layer, suppressing surface recombination and stabilizing the electrode, as shown in Figure 4c. High-resolution transmission electron microscopy (HR-TEM) confirmed successful dopant incorporation into the BiVO_4_ lattice and the formation of well-defined interfaces. Lattice fringes with an interplanar spacing of 0.31 nm corresponded to the (121) plane of highly crystallized BiVO_4_, whereas the top-side fringes with a spacing of 0.19 nm were attributed to the Ni (111) plane, indicating intimate contact between the cocatalyst and the photoanode surface. This multi-step surface modification strategy led to a substantial improvement in PEC performance, achieving a photocurrent density of 3.1 mA/cm^2^ at 1.23 *V_RHE_* under AM 1.5G illumination. These results highlight the effectiveness of combining electronic structure tuning with interfacial engineering to overcome key limitations of BiVO_4_, in particular, poor surface kinetics and charge recombination. The HR-TEM images and photocurrent curves validating the synergistic effect of Ba-doping, HfO_2_ passivation, and NiPt decoration are presented in Figure 4d,e [108].

In conclusion, these studies collectively demonstrate that defect engineering through doping, when coupled with meticulous interfacial engineering, constitutes a powerful approach to significantly enhance the PEC performance of BiVO_4_ photoanodes.

### 4.2. Heterojunction with ETL (Electron Transport Layer)

Despite its favorable optical bandgap and stability under neutral pH conditions, BiVO_4_ photoanodes suffer from poor charge separation and inefficient electron transport, which limit their practical PEC performance. Traditional strategies, such as embedding WO_3_ as a buried electron transport layer, can enhance electron extraction but necessitate thick absorbers, conflicting with the inherently low charge mobility in BiVO_4_.

**Figure 5:**
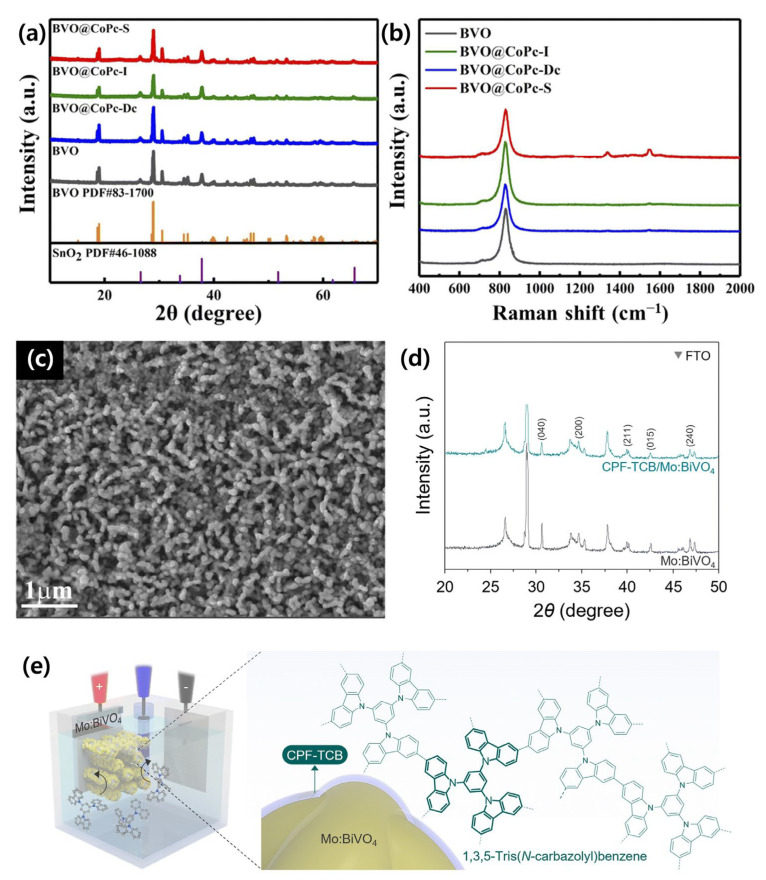
(**a**) XRD patterns; (**b**) Raman spectra and SEM images of the BVO@CoPc-S photoanodes. Reprinted with permission from Ref. [109], Copyright © 2025, Royal Society of Chemistry; (c) SEM image of Mo:BiVO_4_, (**d**) XRD of Mo:BiVO_4_ and CPF-TCB/Mo:BiVO_4_; (**e**) schematic illustration of the electropolymerization of CPF-TCB on Mo:BiVO_4_. Reprinted with permission from Ref. [110], Copyright © 2024, Royal Society of Chemistry.

Yu et al. addressed these limitations by applying an ultrathin Co-phthalocyanine (CoPc) surface layer to the BiVO_4_ photoanode, effectively tuning its surface hydrophilicity. XRD confirmed the formation of monoclinic BiVO_4_ on FTO without observable CoPc peaks, likely due to its low loading and high dispersion, while Raman spectra revealed characteristic C–C and C–N vibrational bands of CoPc, verifying its successful incorporation and stronger interfacial coupling in solvothermally treated samples. The successful incorporation of CoPc and the improved interfacial coupling are supported by the XRD and Raman results shown in Figure 5a,b, where characteristic peaks and morphological changes are clearly observed. This interface modification improved interaction with the electrolyte and suppressed surface recombination, resulting in a photocurrent density of 4.0 mA/cm^2^ at 1.23 *V_RHE_*—approximately 3.1 times higher than that of pristine BiVO_4_—along with enhanced IPCE and operational stability. The ultrathin interface layer overcomes the drawbacks of thick buried ETLs, providing an elegant solution for performance improvement without compromising charge mobility [109]. 

Furthermore, Yang et al. introduced an organic–inorganic heterojunction by conformally coating a conjugated polycarbazole framework (CPF-TCB) onto nanoporous Mo-doped BiVO_4_. The morphology, XRD patterns, and schematic representation of CPF-TCB deposition on Mo:BiVO_4_ are presented in Figure 5c–e, highlighting the conformal coverage and structural features of the hybrid interface. This hole transport layer (HTL) forms a type-II band alignment with BiVO_4_, facilitating efficient hole extraction and suppressing recombination. After loading a NiFeCoO_x_ cocatalyst, the photoanode achieved a record-high water oxidation photocurrent density of 6.66 mA/cm^2^ at 1.23 *V_RHE_*. When integrated into unassisted tandem PEC devices, it delivered solar-to-hydrogen conversion efficiencies of up to 9.02%, setting benchmarks for organic–inorganic hybrid photoelectrodes under operational conditions [110].

Overall, the integration of an external ETL via the carbon shell provides a powerful and scalable strategy to overcome the intrinsic charge transport limitations of oxide semiconductors, offering promising avenues for the development of high efficiency and durable PEC energy conversion systems.

### 4.3. Hole-Transport Layers (HTLs) for BiVO_4_ Photoanodes

Beyond electron transport layers, hole transport layers (HTLs) play a critical role in enhancing the surface charge extraction of BiVO_4_. By creating favorable band offsets for hole transfer, passivating surface trap states that promote interfacial recombination, and offering abundant well-dispersed oxygen evolution sites when coupled with cocatalysts, HTLs directly improve the charge transfer efficiency (*η_trans_*) under water oxidation conditions [111,112,113,114].

**Figure 6:**
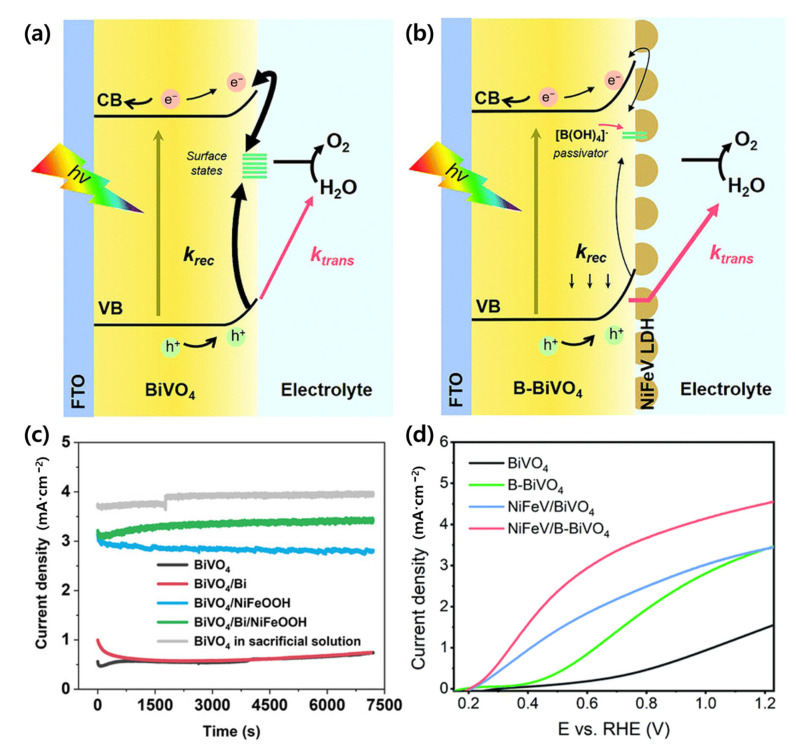
(**a**) Schematic illustration of interfacial hole transport in bare BiVO_4_ photoanode and (**b**) interfacial hole transport pathway in NiFeV/B-BiVO_4_ photoanode. Reprinted with permission from Ref. [113], Copyright © 2021, Royal Society of Chemistry. (**c**) Photocurrent density stability of photoanodes at 0.8 *V_RHE_*. Reprinted with permission from Ref. [111], Copyright © 2022, Wiley-VCH GmbH. (**d**) LSV curves of BiVO_4_, B-BiVO_4_, NiFeV/BiVO_4_, and NiFeV/B-BiVO_4_ photoanodes under AM 1.5G illumination in 1.0 M potassium borate buffer (pH 9.3, scan rate: 10 mV/s). Reprinted with permission from Ref. [113], Copyright © 2021, Royal Society of Chemistry.

Cui et al. demonstrated that inserting a partially oxidized 2D bismuthene layer between BiVO_4_ and NiFeOOH enhanced interfacial band bending, passivated V_O_-related traps, and extended hole lifetime. The interfacial hole transport mechanism and performance enhancement enabled by such interlayers are schematically illustrated in Figure 6a,b. This strategy yielded a 5.8-fold increase in photocurrent compared to bare BiVO_4_, achieving 3.4 ± 0.2 mA/cm^2^ at +0.8 *V_RHE_* and 4.7 ± 0.2 mA/cm^2^ at +1.23 *V_RHE_*, with stable operation under illumination [111].

In another approach, Li et al. employed a serial HTL architecture by sequentially depositing Fe_2_O_3_ (facilitating bulk-to-surface hole transfer) and NiOOH/FeOOH (promoting surface-to-electrolyte transfer) on BiVO_4_. The photocurrent stability and LSV curves of these stepwise HTLs are shown in Figure 6c,d, confirming the substantial improvements in charge transfer efficiency. The resulting photoanode delivered 2.24 mA/cm^2^ at 1.23 *V_RHE_* (≈2.95× over pristine BiVO_4_). At the same potential, *η_bulk_* and *η_surface_* were enhanced by 1.63× and 2.62×, respectively, demonstrating stepwise improvements in charge transfer. When coupled with a commercial Si photovoltaic for self-biasing, the device sustained 2.60 mA/cm^2^, corresponding to a solar-to-hydrogen (STH) efficiency of 3.2%, underscoring the practicality of sequential HTLs [112].

Expanding this concept, Meng et al. reported that NiFeV-LDH deposited on borate-treated BiVO_4_ (B-BiVO_4_) served as an interfacial hole transport/storage layer. The optimized NiFeV/B-BiVO_4_ photoanode achieved 4.6 mA/cm^2^ at 1.23 *V_RHE_* with an early onset potential of 0.2 *V_RHE_*, while retaining ≥80% of the initial photocurrent after 24 h and maintaining ~95% faradaic efficiency. Mechanistic studies revealed a substantial increase in Z_surface (~90% vs. 31% for bare BiVO_4_) and reduced charge transfer resistance (R_ct_), confirming accelerated interfacial hole transfer upon LDH integration [113].

Collectively, these studies highlight that rational HTL design—from 2D bismuthene insertion to serial inorganic HTLs and LDH-based transport/storage layers—offers complementary pathways to lower interfacial barriers, enhance band bending, and provide robust scaffolds for cocatalyst integration, thereby substantially boosting both activity and stability of BiVO_4_ photoanodes.

### 4.4. Cocatalyst and Surface Modification Strategies for BiVO_4_ Photoanodes

A wide range of cocatalyst deposition and surface modification strategies have been developed to improve the PEC performance of BiVO_4_ photoanodes. These approaches primarily aim to accelerate *OER* kinetics, passivate surface trap states, and suppress interfacial charge recombination, thereby enhancing both photocurrent density and operational stability. Table 3 summarizes representative strategies such as dual-layer deposition, ligand-assisted passivation, and facet-selective cocatalyst loading, highlighting their impact on photocurrent density, onset potential, Faradaic efficiency, and durability.

**Figure 7:**
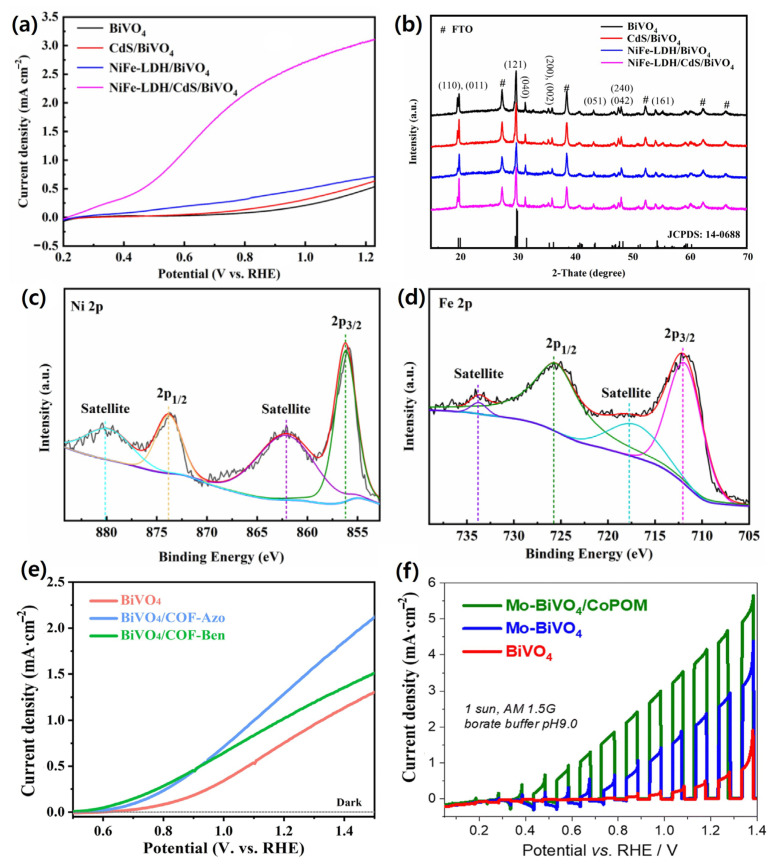
(**a**) LSV curves and (**b**) XRD patterns of NiFe-LDH/CdS/BiVO_4_, NiFe-LDH/BiVO_4_, CdS/BiVO_4_, and pristine BiVO_4_ photoanodes measured in 0.5 M Na_2_SO_4_ electrolyte (pH 6.1) at 1.23 *V_RHE_*. (**c**,**d**) XPS spectra of Ni 2p and Fe 2p, respectively. Reprinted with permission from Ref. [49], Copyright © 2024, MDPI. (**e**) LSV and *OER* polarization curves of the photoanodes measured without illumination Reprinted with permission from Ref. [124], Copyright © 2024, Royal Society of Chemistry. (**f**) LSV curves recorded under intermittent AM 1.5 G illumination in 0.5 M borate buffer (pH 9.0) with a cathodic scan rate of 10 mV/s. Reprinted with permission from Ref. [125], Copyright © 2024, Royal Society of Chemistry.

Among these strategies, Dong et al. reported a dual modification approach of coupling CdS nanoparticles with NiFe-LDH nanosheets on BiVO_4_. XRD confirmed the monoclinic scheelite phase of BiVO_4_, while the absence of distinct CdS and NiFe-LDH peaks was attributed to the low CdS loading and amorphous nature of NiFe-LDH. XPS verified the coexistence of CdS and NiFe-LDH through Ni^2+^ and Fe^3+^ peaks, and SEM/HRTEM revealed a defect-rich amorphous nanosheet structure providing abundant active sites. The CdS nanoparticles broadened the light absorption range and facilitated charge transfer, while the NiFe-LDH overlayer functioned as a cocatalyst to accelerate *OER* kinetics and passivate surface traps. This synergistic interface engineering significantly improved charge separation and transport properties, resulting in a photocurrent density of 3.1 mA cm^−2^ at 1.23 V_RHE—about 5.8 times higher than bare BiVO_4_—and excellent stability under continuous illumination. The enhanced photocurrent density, crystallographic features, and XPS confirmation of the cocatalyst loading are shown in Figure 7a–d [49].

Beyond inorganic systems, organic framework materials such as MOFs and Covalent organic frameworks (COFs) offer a molecularly tunable platform with intrinsically high surface area and porosity, enabling precise control over catalytic site density and efficient mass transport. Guo et al. reported the in situ growth of COF–Azo and COF–Ben on BiVO_4_, yielding hybrids with reduced charge transfer resistance, lower onset potential, and higher photocurrent densities compared to pristine BiVO_4_. The improved *OER* activity of the COF–Azo modified electrode is evidenced by the polarization and LSV curves in Figure 7e. EIS confirmed enhanced interfacial kinetics, and the intimate COF–BiVO_4_ heterojunction facilitated efficient hole extraction and suppressed recombination, leading to markedly improved *OER* activity [124]. Similarly, Feng et al. demonstrated that combining Mo doping with cobalt polyoxometalate (CoPOM) deposition effectively enhanced bulk charge transport, lowered *OER* overpotential, and delivered significantly higher photocurrent densities with improved operational stability. The photocurrent enhancement and stability of the CoPOM-modified BiVO_4_ are illustrated in Figure 7f [125]. Collectively, these results demonstrate that both inorganic cocatalysts/overlayers and MOF/COF-based organic frameworks provide complementary strategies to overcome sluggish surface kinetics and interfacial recombination in BiVO_4_ photoanodes, ultimately enabling high efficiency and durable PEC water splitting devices.

### 4.5. Light Absorption Efficiency

BiVO_4_ possesses a bandgap of approximately 2.4 eV, enabling the absorption of photons with wavelengths ranging from 300 to 520 nm [126]. Under AM 1.5G solar illumination, the theoretical maximum photocurrent density (*J_max_*) is 7.5 mA/cm^2^ [20]. In practice, however, optical losses due to light refraction and reflection reduce effective light absorption, yielding an actual theoretical photocurrent density of only 4.45 mA/cm^2^ [127]. This discrepancy highlights that light absorption efficiency is a critical factor limiting the PEC performance of BiVO_4_-based photoanodes.

Enhancing light absorption is, therefore, essential for improving PEC activity. Effective strategies include constructing heterostructures and integrating optical elements into the BiVO_4_ architecture, both of which can strengthen photon–matter interactions [16,128]. These approaches increase photon utilization and, consequently, improve the overall performance of the photoanode.

#### 4.5.1. Band Edge Engineering

Reducing the bandgap of semiconductor photocatalysts is an established strategy to broaden their light absorption spectrum, potentially extending it into the near-infrared region. Among various approaches, defect engineering via doping is particularly effective for BiVO_4_, as it introduces defect levels within the band structure, modifies the electronic configuration, shifts the band edges, and enhances light absorption. Careful control of dopants and defect states allows the bandgap to be narrowed without compromising charge transport, resulting in improved PEC performance.

**Figure 8:**
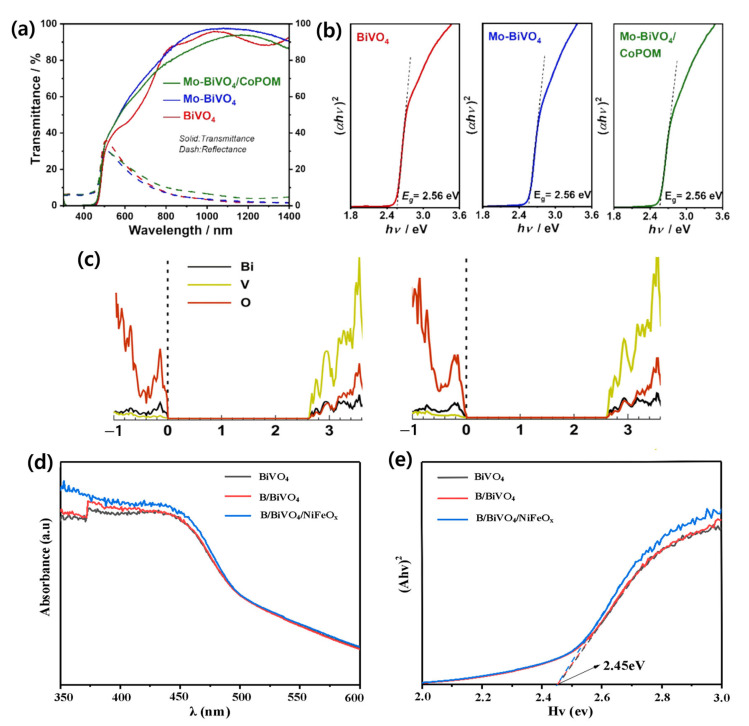
(**a**) UV–Vis absorption spectra of Mo-BiVO_4_/CoPOM, Mo-BiVO_4_ and pristine BiVO_4_ photoanodes with (**b**) Tauc plots for bandgap determination. Reprinted with permission from Ref. [125], Copyright © 2024, Royal Society of Chemistry. (**c**) Projected density of states (PDOS) comparison for undoped t-BiVO_4_ (**left**) and m-BiVO_4_ (**right**) host structures. Reprinted with permission from Ref. [129], Copyright © 2024, Royal Society of Chemistry. (**d**) UV–Vis absorption spectra of BiVO_4_, B/BiVO_4_, and B/BiVO_4_/NiFeO_x_ photoanodes and (**e**) corresponding Tauc plots. Reprinted with permission from Ref. [130], Copyright © 2025, MDPI.

Fan Feng et al. systematically investigated the synergistic effects of Mo doping and surface modification with a cobalt polyoxometalate (CoPOM) on BiVO_4_ photoanodes. UV–Vis absorption spectra showed similar absorption onsets of around 515 nm for all samples, and Tauc plot analysis indicated bandgap energies of ~2.56 eV, confirming that Mo doping and CoPOM deposition do not alter the fundamental absorption edge or introduce significant parasitic light absorption. This suggests that the observed performance enhancement originates primarily from improved charge transport and interfacial catalytic activity rather than changes in light-harvesting properties. Photocurrent–potential curves showed that Mo doping alone increased the photocurrent from ~0.65 to ~2.53 mA/cm^2^ at 1.23 *V_RHE_*, while the combination of Mo–BiVO_4_/CoPOM further enhanced it to ~4.32 mA/cm^2^. The absorption spectra, Tauc plots, and density of states analysis supporting these results are shown in Figure 8a–c. As a result, this represents a ~6.6-fold improvement over pristine BiVO_4_ and ~1.7-fold compared to Mo-doped BiVO_4_ alone. The enhancement is attributed to improved conductivity through Mo doping and accelerated water oxidation kinetics via CoPOM catalysis. Electrochemical impedance spectroscopy (EIS) confirmed a significant reduction in charge transfer resistance, while the optical appearance of the electrodes validated enhanced visible light response [125].

Similarly, Elnagar et al. demonstrated that Mo-doped BiVO_4_ modified with a CoPOM surface layer exhibited a slight bandgap narrowing and extended light absorption into the longer-wavelength visible region [129]. The UV–Vis absorption spectra and Tauc plots confirming this bandgap narrowing are presented in Figure 8d,e. This, combined with surface modification, resulted in synergistic improvement in PEC performance.

Furthermore, Zhou et al. reported that co-doping BiVO_4_ with Mo and Ni effectively narrows the bandgap and enhances light-harvesting capability. UV–Vis spectra showed a red-shift in the absorption edge, corresponding to a bandgap reduction of approximately 0.15–0.20 eV compared to pristine BiVO_4_. The co-doped photoanode exhibited a photocurrent density of ~3.6 mA/cm^2^ at 1.23 *V_RHE_*, over 40% higher than undoped BiVO_4_. EIS analysis confirmed improved charge transfer resistance, and the color change in the electrodes reflected the enhanced visible light response. These results indicate that band-edge tuning via Mo–Ni co-doping effectively extends light absorption and boosts PEC activity [130].

These findings collectively confirm that band-edge engineering through doping and defect creation remains a promising and actively evolving strategy to enhance the PEC performance of BiVO_4_ photoanodes in solar water splitting.

#### 4.5.2. Constructing Heterojunction Structure

Constructing heterojunctions is an effective strategy to enhance the light-harvesting efficiency (*LHE*) of BiVO_4_, suppress charge recombination, and accelerate *OER* kinetics. By coupling BiVO_4_ with suitable semiconductors or cocatalysts, researchers have developed advanced heterostructures that significantly improve PEC water splitting performance.

Seo et al. demonstrated the effectiveness of a p–n heterojunction between BiVO_4_ and PbS quantum dots (QDs), combined with a ZnS overlayer, for enhanced PEC hydrogen production [54]. Nanoporous BiVO_4_ films were prepared via electrodeposition, followed by PbS QD sensitization using the successive ionic layer adsorption and reaction (SILAR) method. The PbS QDs extended light absorption into the near-infrared region and improved charge separation through the p–n junction interface. Subsequently, a conformal ZnS overlayer was deposited to passivate surface defects on the QDs, reducing non-radiative recombination.

**Figure 9:**
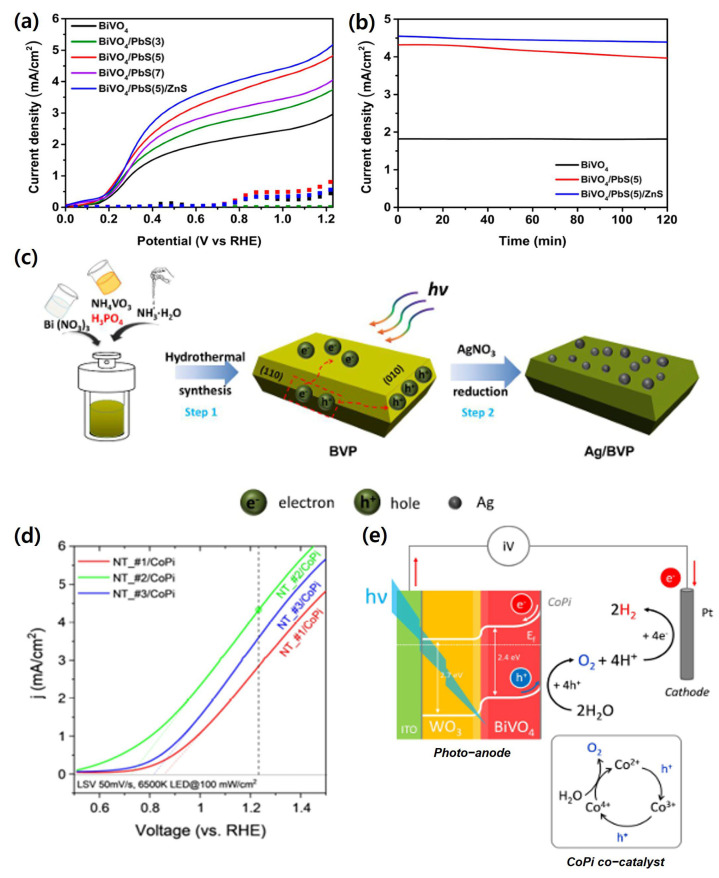
(**a**) *J*–*V* curves and (**b**) chronoamperometric curves (at 1.23 *V_RHE_*) of bare BiVO_4_, BiVO_4_/PbS(n) QDs, and BiVO_4_/PbS(5) QDs/ZnS photoanodes (n: the number of PbS SILAR cycles). Reprinted with permission from Ref. [56], Copyright © 2023, MDPI. (**c**) Scheme of synthetic route of dually modified BiVO_4_ photocatalyst. Reproduced from Ref. [131], under Creative Commons CC BY license. (**d**) LSV diagrams under illumination with blue light for NT_#1/CoPi, NT_#2/CoPi and NT_#3/CoPi electrodes. (**e**) Band diagram describing the fate of photoelectron and photohole upon light absorption by the BiVO_4_ semiconductor. Reproduced from Ref. [132], under Creative Commons CC BY license.

As a result, the photocurrent density at 1.23 *V_RHE_* increased from 2.92 mA/cm^2^ for bare BiVO_4_ to 4.88 mA/cm^2^ after PbS QD sensitization, and further to 5.19 mA/cm^2^ with the ZnS overlayer. The *J*–*V* and stability curves of these heterostructured electrodes are shown in Figure 9a,b, confirming the improved photocurrent and durability. EIS confirmed a substantial reduction in charge transfer resistance (R_ct_), indicating enhanced surface kinetics. Stability tests over 2 h revealed retention rates of 91.24% for BiVO_4_/PbS and 96.20% for BiVO_4_/PbS/ZnS, emphasizing the role of passivation layers in maintaining operational durability. This study highlights that combining heterojunction engineering with surface passivation can simultaneously maximize efficiency and stability in BiVO_4_-based PEC systems.

Similarly, Zhang et al. reported highly efficient BiVO_4_ single-crystal nanosheets modified via phosphorus doping and selective Ag decoration. The synthetic route and structural modification strategy are schematically illustrated in Figure 9c. This dual modification generated oxygen vacancies and improved charge migration pathways, leading to a ~2.8-fold increase in methylene blue degradation compared to pristine BiVO_4_ under visible light irradiation [131].

Li et al. fabricated nanostructured BiVO_4_/Co-Pi heterostructures using a vanadium-infused interaction method. The incorporation of the Co-Pi cocatalyst facilitated efficient hole extraction and prolonged carrier lifetime, enabling a photocurrent density of 2.9 mA/cm^2^ at 1.23 *V_RHE_* under simulated solar illumination [47]. These results underscore the effectiveness of heterojunction construction and cocatalyst integration for enhancing the PEC performance of BiVO_4_ photoanodes.

In another example, Gil-Rostra et al. developed ITO/WO_3_/BiVO_4_/CoPi multishell nanotube arrays using a soft-templating technique [132]. The intimate WO_3_/BiVO_4_ interface generated an internal electric field that enhanced charge separation, while the CoPi overlayer improved surface *OER* kinetics. In Figure 9d, LSV curves confirmed a substantial increase in photocurrent upon CoPi deposition, and the band diagram illustrates the overall charge transport pathway: photoholes generated in BiVO_4_ migrate to the surface to drive *OER*, whereas photoexcited electrons are efficiently funneled through the WO_3_ shell and ITO layer to the external circuit. This heterostructured architecture enables efficient light absorption within BiVO_4_, while the nanoscale tubular design shortens the electron diffusion path, thereby minimizing ohmic losses and maximizing charge collection. The corresponding band diagram describing the fate of photogenerated carriers is presented in Figure 9e. The resulting hierarchical electrode exhibited outstanding stability and PEC performance under one-sun illumination.

These studies collectively demonstrate that combining heterojunction formation with surface modification strategies can simultaneously maximize optical absorption, charge separation, and operational stability of BiVO_4_-based photoanodes, paving the way toward efficient solar fuel generation.

#### 4.5.3. Optics-Based Elements

Tuning the morphological features of photocatalysts provides an effective strategy to modulate light scattering and reflection, thereby enhancing overall light absorption and improving catalytic performance [133]. Three-dimensional (3D) inverse opal architectures, in particular, have emerged as a powerful design due to their highly ordered macro–mesoporous networks and substantially increased specific surface areas [134].

A recent study reported the fabrication of a 3D core–shell inverse opal photoanode composed of FTO/TiO_2_/BiVO_4_, using a combination of atomic layer deposition and electrodeposition techniques [135]. This configuration creates a uniform opal framework that facilitates rapid electron transport and improved light management, enabling the photoanode to achieve a photocurrent density of 4.11 mA/cm^2^ at 1.23 *V_RHE_*, significantly outperforming comparable planar structures.

**Figure 10:**
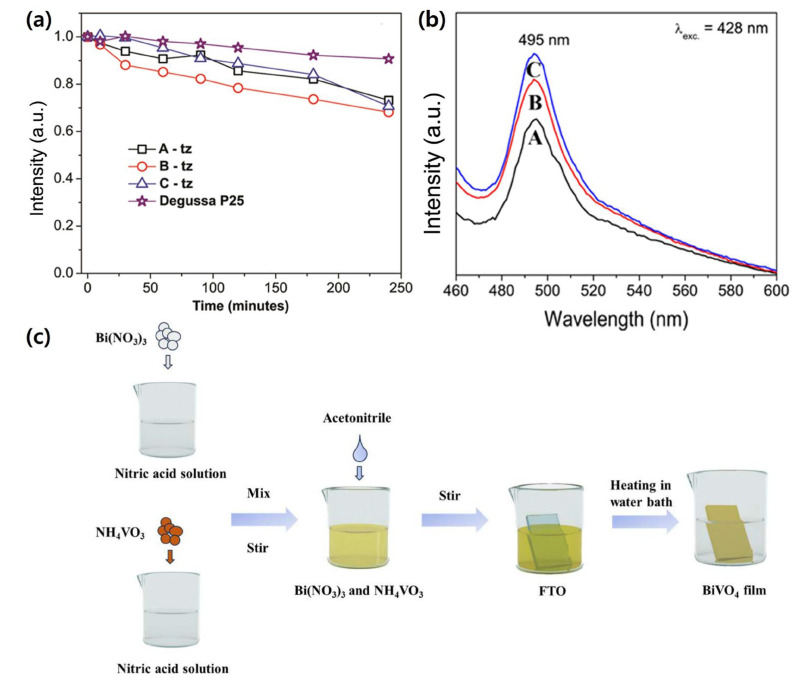
(**a**) Photoluminescent emission spectra of tz-BiVO_4_ nanoparticles in ethylene glycol, (**b**) photodegradation curves of MO solution (5 mg/L) by different tz-BiVO_4_ samples and Degussa P25 (1 g/L) under UV-Vis lighting of MO in the presence of the tz-BiVO_4_ photocatalyst. Reprinted with permission from Ref. [136], Copyright © 2025, MDPI. (**c**) Schematic illustration of the synthesis and characterizations of BiVO_4_ catalytic films. Reprinted with permission from Ref. [137], Copyright © 2024, MDPI.

For example, Marinković et al. synthesized zircon-type BiVO_4_ nanoparticles (2–8 nm) via a facile colloidal route. The nanosized BiVO_4_ exhibited a tetragonal zircon structure with enhanced optical absorption and a significantly enlarged surface-to-volume ratio. These features led to superior photocatalytic degradation of methyl orange compared with commercial TiO_2_, underscoring the potential of ultrafine BiVO_4_ nanoparticles for efficient light harvesting and environmental remediation The photoluminescence spectra and photodegradation performance confirming these results are presented in Figure 10a,b [136].

Similarly, Zhao et al. demonstrated that controlling the crystal orientation of BiVO_4_ thin films has a critical impact on PEC behavior. BiVO_4_ films preferentially oriented along the [010] direction exhibited markedly improved carrier separation and mobility compared to [119]-oriented or randomly oriented samples. The synthesis process and characterization of these catalytic films are schematically illustrated in Figure 10c. As a result, the [010]-oriented BiVO_4_ delivered a photocurrent density of 0.2 mA/cm^2^ at 1.21 *V_RHE_*, outperforming [119]-oriented (0.056 mA/cm^2^) and random films (0.11 mA/cm^2^) [137]. Despite these significant improvements in light absorption, optimizing light utilization remains a key challenge in advancing the performance of BiVO_4_ photoanodes.

### 4.6. Photogenerated Charge Separation Efficiency (η_sep_)

The primary bottleneck in the PEC efficiency of BiVO_4_ photoanodes is the rapid recombination of photogenerated electron–hole pairs. This limitation is further exacerbated by the low carrier mobility of BiVO_4_, which is approximately 10^−2^ cm^2^/V·s, resulting in substantial charge recombination before the charges can reach the electrode surface [138]. Enhancing charge migration is, therefore, essential to unlock the full PEC potential of BiVO_4_.

To mitigate recombination, extensive strategies have been developed to improve carrier transport and separation. These include elemental doping to modulate carrier density and defect states, construction of heterojunctions to establish favorable band alignments, and interface engineering with cocatalysts to accelerate surface charge transfer. Additionally, defect engineering, such as introducing oxygen vacancies, can further promote charge separation by creating internal electric fields or shallow trap states that reduce recombination.

Representative examples are summarized in Table 4, which highlights the PEC performance and *η_sep_* of selected BiVO_4_-based photoanodes. For instance, the introduction of a WO_3_/S:Bi_2_O_3_/(Ga,W):BiVO_4_/Co–Pi interface structure significantly improved photocurrent density through optimized charge transfer pathways [135], while oxygen vacancy-engineered BiVO_4_ (Ov-BiVO_4_) achieved an outstanding *η_sep_* of 94% with a photocurrent density of 6.29 mA/cm^2^ [139]. These results collectively demonstrate that tailored modifications in BiVO_4_ can directly translate to higher charge separation efficiency and overall PEC activity.

Taken together, these findings underscore that *η_sep_* serves as a crucial performance metric in evaluating BiVO_4_-based photoanodes. Continued progress in tuning bulk properties, interface chemistry, and defect states has already shown remarkable improvements, but further breakthroughs will likely depend on synergistically integrating multiple strategies.

Therefore, improving the charge separation efficiency of BiVO_4_ requires a comprehensive consideration of multiple strategies, including the utilization of internal electric fields and the modulation of conductivity and carrier density. The following section will examine these representative approaches in detail, with a focus on their underlying mechanisms and demonstrated performance.

#### 4.6.1. Utilizing in Internal Electric Field

A practical approach to overcome the intrinsically low charge separation efficiency (*η_sep_*) of BiVO_4_ is the construction of heterojunctions that induce a built-in internal electric field (IEF). This IEF bends the energy bands at the interface, directing electrons and holes in opposite directions and thereby extending carrier lifetimes. As a result, the overall PEC water splitting performance is significantly enhanced.

**Figure 11:**
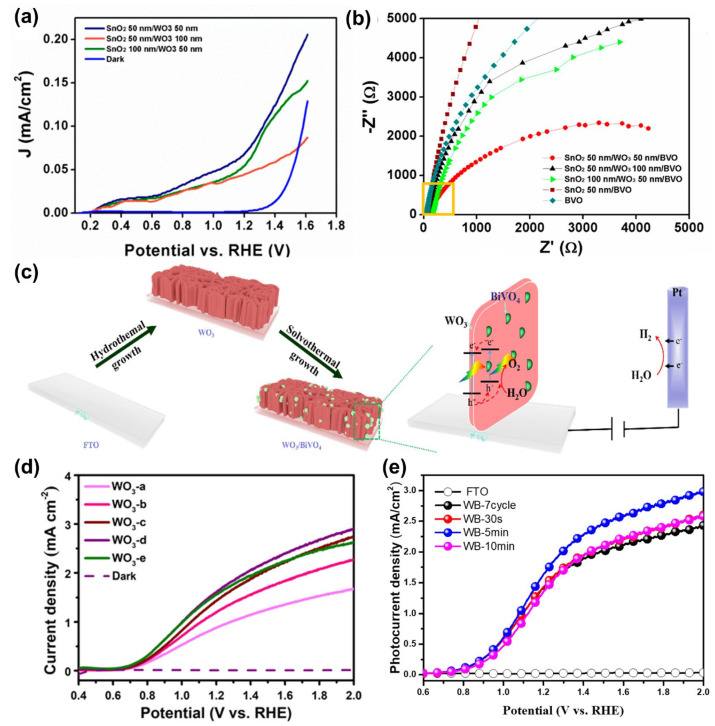
(**a**) LSV of SnO_2_/WO_3_ photoanode measured using a three-electrode configuration set up in aqueous phosphate buffer (pH 7.0) with 0.5 M Na_2_SO_3_, (**b**) EIS of SnO_2_/WO_3_/BiVO_4_. Reprinted with permission from Ref. [147], Copyright © 2028, MDPI. (**c**) Schematic illustration of the formation and the PEC water oxidation mechanism of the WO_3_/BiVO_4_ arrays. The red flakes represent WO_3_ and the green particles represent BiVO_4_ (**d**) LSV curves of the photoelectrode. Reprinted with permission from Ref. [148], Copyright © 2024, MDPI. (**e**) *I*–*V* curves for FTO, WB-7 cycle, WB-30 s, WB-5 min, and WB-10 min samples. Reprinted with permission from Ref. [149], Copyright © 2020, MDPI.

For example, Bhat et al. constructed a triple planar SnO_2_/WO_3_/BiVO_4_ heterojunction. The staggered (type-II) band alignment, along with the SnO_2_ hole-blocking layer, facilitated interfacial charge separation and electron transport, yielding a photocurrent density of ~2.01 mA/cm^2^ at 1.23 *V_RHE_* under front illumination, with remarkably high IPCE values of ~90% (front) and ~80% (back) at 400 nm. The photocurrent response and impedance characteristics of this triple planar heterojunction are presented in Figure 11a,b. The authors attributed this enhancement to efficient interfacial separation and reduced recombination under front illumination, consistent with the effect of a built-in field across the multilayer junction [147].

Similarly, Liang et al. fabricated surface-dispersed WO_3_/BiVO_4_ heterojunction arrays (BiVO_4_-nanoparticle@WO_3_-nanoflake). Decorating WO_3_ nanoflakes uniformly with ~20–50 nm BiVO_4_ nanoparticles increased the effective junction area and mitigated interfacial hole accumulation, thereby enhancing charge separation. The schematic formation process and corresponding LSV curves of these heterojunction arrays are shown in Figure 11c,d. The resulting composite achieved a photocurrent density of 3.53 mA/cm^2^ for PEC water oxidation—approximately twice that of WO_3_ alone—while maintaining good stability, indicating a robust IEF over the nanojunction network [148].

In another optics-plus interface example, Cao et al. engineered an FTO/WO_3_/BiVO_4_ heterojunction and applied a mild NaOH post-treatment to modify the BiVO_4_ surface chemistry. The heterojunction provided an interfacial driving force for carrier separation, while the surface treatment accelerated water oxidation kinetics, together yielding ~1.75 mA/cm^2^ at 1.23 *V_RHE_*—more than double that of WO_3_,as shown in Figure 11e [149].

Collectively, these studies demonstrate that establishing heterointerfaces (SnO_2_/WO_3_/BiVO_4_; WO_3_/BiVO_4_) and tuning surface chemistry can create or strengthen internal electric fields, which (i) promote directional carrier migration, (ii) suppress interfacial recombination, and (iii) enhance photocurrent and IPCE without the need for precious metal cocatalysts.

#### 4.6.2. Enhancing Electrical Conductivity and Carrier Concentration

Enhancing the electrical conductivity of BiVO_4_ has emerged as an important strategy to improve its intrinsic material properties [150]. Doping, in particular, is widely recognized as an effective means of tuning the electronic structure and thereby increasing conductivity [151]. Both metal and nonmetal dopants have been successfully incorporated into BiVO_4_ for this purpose [152]. Among them, tungsten (W) and molybdenum (Mo) are especially effective: substituting V^5+^ ions with W^6+^ or Mo^6+^ ions significantly enhance the electronic conductivity of BiVO_4_ [153].

**Figure 12:**
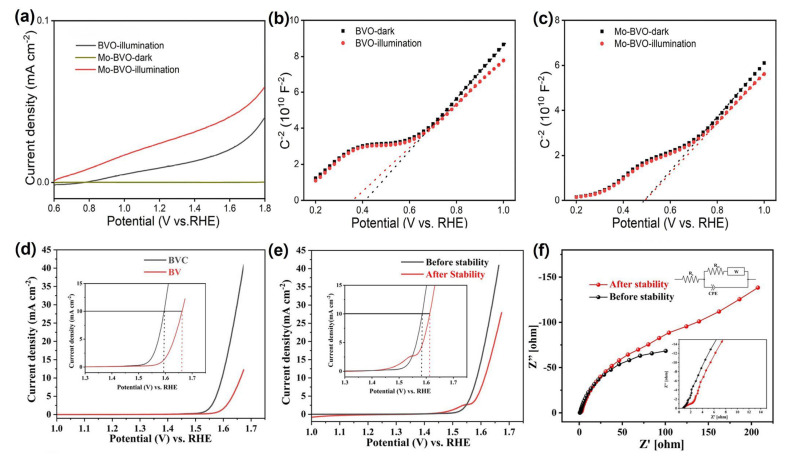
(**a**) LSV curves of as-prepared BiVO_4_-based samples measured both in the dark and under illumination (λ_exc_ = 435 nm, 100 mW/cm^2^) in 0.1 M phosphate buffer and 0.5 M Na_2_SO_3_ solutions, showing enhanced photocurrent under illumination. (**b**,**c**) Mott–Schottky plots of pristine BVO and Mo-doped BVO, respectively, highlighting changes in donor density and flat-band potential upon Mo incorporation. Reprinted with permission from Ref. [154], Copyright © 2023, Royal Society of Chemistry. (**d**) LSV curves comparing BiVO_4_ and BiVO_4_/carbon composite electrodes under PEC conditions, (**e**) LSV curves for *OER* measured before and after stability testing at a scan rate of 1 mV/s, demonstrating the effect of stability treatment, and (**f**) EIS Nyquist plots of the electrodes before and after CA stability testing, revealing changes in charge transfer resistance. Reprinted with permission from Ref. [155], Copyright © 2025, OAE Publishing Inc.

Enhancing the electrical conductivity of BiVO_4_ is a critical strategy to facilitate efficient charge transport and suppress recombination losses during PEC water splitting. Improved conductivity not only enables faster carrier movement but also contributes directly to higher photocurrent generation. Wu et al. demonstrated that Mo doping in BiVO_4_ significantly lowers the interfacial charge transfer resistance by 2–3 orders of magnitude under illumination. This finding indicates that interfacial kinetics, rather than bulk resistance, dominate the PEC performance of pristine BiVO_4_. As a result, the enhanced conductivity and accelerated charge transfer led to a remarkable increase in photocurrent density, reaching values ~2.7 times higher than those of undoped BiVO_4_ at 1.23 *V_RHE_*. Figure 12a–c present the LSV curves and Mott–Schottky plots, confirming the enhanced donor density and reduced resistance [154].

Similarly, Chaudhari et al. reported that introducing a conductive carbon network into BiVO_4_ through MOF-derived Bi–V–O/carbon composites (BVC) effectively reduced charge transport resistance and enlarged the electrochemically active surface area, leading to enhanced PEC water oxidation activity and additional applicability in energy storage systems. The corresponding LSV, stability, and EIS results supporting these improvements are shown in Figure 12d–f [155].

Taken together, these results underscore that strategies improving the electrical conductivity of BiVO_4_—whether through cation doping or conductive composite engineering—consistently enhance PEC performance by lowering resistance, increasing carrier density, and enabling more efficient charge extraction.

### 4.7. Charge Transfer Efficiency (η_trans_)

Enhancing the charge transfer efficiency *η_trans_* at the photoanode/electrolyte interface is crucial for realizing efficient PEC performance in BiVO_4_. Although photogenerated holes in BiVO_4_ can reach the electrode surface to drive the *OER*, a substantial fraction is often lost through recombination with electrons during transport or at the interface. To assess this limitation, *η_trans_* is generally determined by comparing the photocurrent densities measured in the presence and absence of a hole scavenger such as Na_2_SO_3_, which suppresses surface recombination and reflects the ideal case of complete hole utilization. The efficiency can be expressed as

(8) $$\eta_{transfer}\left(\%\right)=\frac{J_{H_{2}O}}{J_{{Na}_{2}{SO}_{3}}}\times 100$$

where *J_H_*_2*O*_ represents the photocurrent density obtained in water oxidation, and *J_Na_*_2*SO*3_ corresponds to the photocurrent density measured with Na_2_SO_3_ present.

This evaluation emphasizes that the sluggish surface reaction kinetics of BiVO_4_ remain a major bottleneck for water oxidation. Accordingly, considerable effort has been devoted to loading suitable cocatalysts onto BiVO_4_, which not only suppress surface recombination but also lower the reaction overpotential. These strategies have been consistently reported to enhance *η_trans_*, resulting in improved photocurrent densities and overall PEC water splitting efficiency.

#### Co-Catalysts Based on Metal (Oxy) Hydroxides

Incorporating metal (oxy)hydroxide (M-OOH) cocatalysts, such as FeOOH and NiFeOOH, onto BiVO_4_-based photoanodes has emerged as one of the most effective approaches to enhance charge transfer efficiency (*η_trans_*). These cocatalysts supply abundant active sites, suppress surface recombination, and accelerate water oxidation kinetics [156].

**Figure 13:**
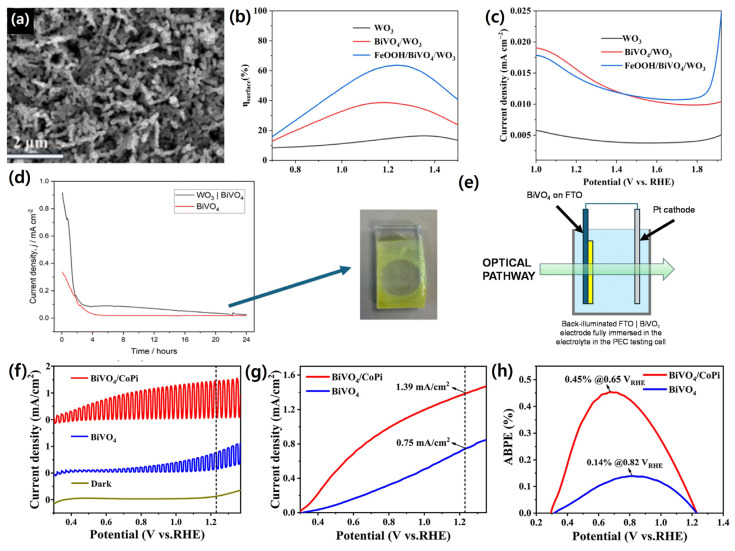
(**a**) SEM surface morphology images of BiVO_4_/WO_3_ photoanodes, (**b**) surface photovoltage efficiency (*η_surface_*) curves, and (**c**) dark current curves of WO_3_, BiVO_4_/WO_3_, and FeOOH/BiVO_4_/WO_3_ electrodes. Reprinted with permission from Ref. [157], Copyright © 2024, MDPI. (**d**) Chronoamperometric stability tests of BiVO_4_ and WO_3_|BiVO_4_ electrodes at 1.23 *V_RHE_* in 0.1 M KPi (pH 7) and photograph showing visible degradation on the irradiated area of WO_3_|BiVO_4_ after 24 h testing, (**e**) schematic optical pathway of the PEC cell used for preliminary measurements of WO_3_|BiVO_4_ electrodes. Reprinted with permission from Ref. [158], Copyright © 2025, Royal Society of Chemistry. (**f**,**g**) *J*–*V* curves of BiVO_4_ and BiVO_4_/CoPi photoanodes under dark conditions and simulated sunlight illumination, respectively, and (**h**) corresponding ABPE values. Reprinted with permission from Ref. [159], Copyright © 2023, MDPI.

For instance, Li et al. fabricated a micro–nanostructured FeOOH/BiVO_4_/WO_3_ photoanode via a combination of hydrothermal, electrodeposition, and impregnation methods. The resulting heterostructure exhibited a photocurrent density of ~2.04 mA/cm^2^ at 1.23 *V_RHE_*, nearly doubling that of the FeOOH-free counterpart (~1.09 mA/cm^2^), along with a positive shift in onset potential from 0.80 to 0.60 *V_RHE_*. The SEM morphology, surface photovoltage, and dark current characteristics supporting these improvements are shown in Figure 13a–c [157].

Similarly, Creasey et al. constructed a WO_3_/BiVO_4_/NiFeOOH photoanode using a scalable aerosol-assisted CVD process. The incorporation of NiFeOOH not only stabilized the heterojunction but also suppressed BiVO_4_ dissolution, enabling a stable photocurrent of 1.75 mA/cm^2^ at 1.23 *V_RHE_* that was sustained for 24 h under one-sun illumination, as shown in Figure 12d,e [158].

Another study by Li et al. reported BiVO_4_/CoPi electrodes prepared through in situ electrodeposition. The CoPi layer increased photocurrent by ~1.8× relative to bare BiVO_4_, achieving 1.39 mA/cm^2^ at 1.23 *V_RHE_*, while simultaneously enhancing applied-bias photon-to-current efficiency (ABPE) and reducing onset potential—highlighting its role in effective passivation and charge separation. The *J*–*V* curves and ABPE values of the BiVO_4_/CoPi photoanodes are illustrated in Figure 13f–h [159].

Collectively, these findings confirm that M-OOH cocatalyst coatings on BiVO_4_ or BiVO_4_-based heterostructures significantly promote charge transfer, reduce onset potential, enhance stability, and thereby improve overall PEC performance.

### 4.8. Stability

The operational stability of BiVO_4_ photoanodes is a key factor for their practical application, as maintaining a consistent photocurrent over extended periods is essential. Photocorrosion, caused by undesirable redox reactions between photogenerated electrons and holes within the BiVO_4_ lattice, can significantly reduce charge extraction efficiency. This degradation is further exacerbated by light-induced leaching of V^5+^ ions, which distorts the lattice and causes long-term structural damage. Recent studies have highlighted two primary strategies to mitigate these effects: the incorporation of protective surface layers and the optimization of electrolyte composition, both of which are discussed in the following sections.

#### 4.8.1. Protection Layer Incorporation

Photocorrosion in BiVO_4_ photoanodes primarily arises from photogenerated holes reacting with surface atoms or via light-assisted dissolution processes, resulting in structural degradation and reduced stability. A practical approach to mitigate these effects is the application of a protective layer, which physically blocks corrosive interactions while still allowing hole transfer to support *OER*.

**Figure 14:**
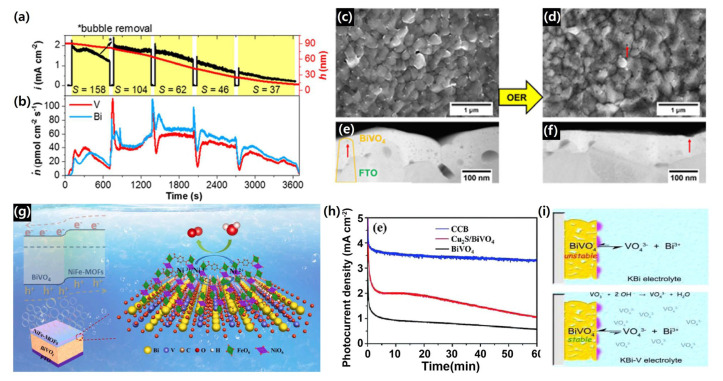
(**a**) Time profiles of photocurrent density i (black), film thickness h (red) the mark * indicates the bubble removal points, (**b**) dissolution rates of BiVO_4_ held at 1.6 *V_RHE_* in borate, (**c**,**e**) top view of scanning electron microscopy (SEM), (**d**,**f**) cross-section view of scanning transmission electron microscopy (STEM). Reproduced from Ref. [160], under Creative Commons CC BY license. (**g**) Schematic diagram of the water oxidation of NiFe-MOFs/BiVO_4_. (**h**) Stability of Co(OH)*_x_*/p-Cu_2_S/n-BiVO_4_ series photoanode in 0.5 M KPi. (**i**) Schematic illustration of the restraint of photocorrosion in KBi–V. Reprinted with permission from Ref. [161], Copyright © 2023, Wiley-VCH GmbH.

For example, Zhang et al. systematically examined the operando photostability of BiVO_4_ in near-neutral electrolytes using a scanning flow cell, directly tracking dissolution during light-driven *OER*. They demonstrated that the dissolution rate is strongly dependent on the electrolyte composition (borate > phosphate > citrate), and that citrate provides kinetic protection via hole scavenging. Distinct dissolution potentials for Bi and V were identified, and time-resolved measurements revealed that photocurrent and dissolution can evolve independently, highlighting that surface chemistry control is essential for achieving long-term stability. These findings underscore the need for barrier or passivation layers to decouple corrosion from charge extraction on BiVO_4_ surfaces. Figure 14a–f provides direct visual confirmation of these dynamics, coupling photocurrent/thickness profiles with dissolution rate measurements and post-mortem morphology analyses under borate and phosphate conditions [160].

Similarly, Wang et al. reviewed interface regulation strategies for BiVO_4_, emphasizing artificial protection layers—such as ultrathin ALD oxides (TiO_2_, Al_2_O_3_) and organic/organic–inorganic interlayers—that suppress photocorrosion while maintaining hole transfer. The review highlighted the design principles for these coatings, which include (i) blocking electrolyte attack, (ii) passivating surface traps, and (iii) forming favorable band alignment for *OER* catalysis, thereby lowering interfacial resistance, shifting onset potentials, and improving operational durability under AM 1.5G illumination. Representative protection strategies and schematic illustrations of stabilized BiVO_4_ electrodes are shown in Figure 14g–i. [161].

Collectively, these examples demonstrate that protective layers—whether polymeric or ALD-oxide—are central to stabilizing BiVO_4_ photoanodes, mitigating operando dissolution, and sustaining interfacial hole transport necessary for efficient oxygen evolution, ultimately enabling durable and high-performance PEC devices.

#### 4.8.2. Dual-Protection Strategies for Durable BiVO_4_ Photoanodes

Photocorrosion and dissolution of BiVO_4_—especially the leaching of V^5+^ ions—represent critical challenges to achieving long-term PEC stability. A practical approach to mitigate these effects involves tuning the electrolyte composition to suppress dissolution and stabilize the surface chemistry.

**Figure 15:**
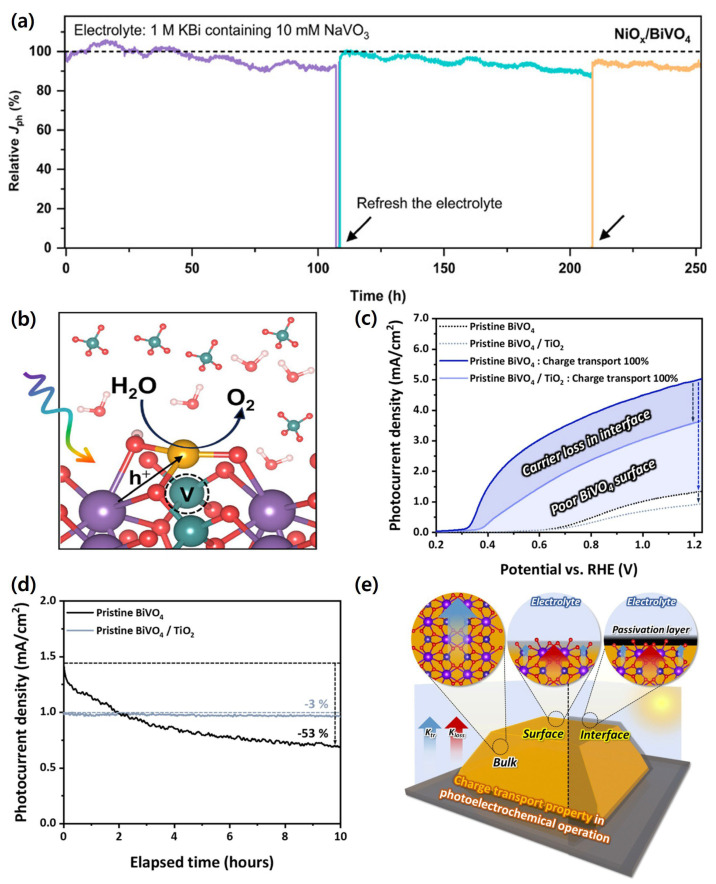
(**a**) *J*–*t* curve of the NiO_x_/BiVO_4_ at 1.23 *V_RHE_*. The electrolyte was replaced at 108 and 208 h, respectively. (**b**) Schematic illustration of the proposed anticorrosion mechanism of NiO_x_/BiVO_4_. Reprinted with permission from Ref. [162], Copyright © 2023, American Chemical Society. (**c**) PEC performances of P-BiVO_4_ (black line) and P-BiVO_4_/TiO_2_ (sky-blue line) photoanodes in general potassium borate electrolyte (dashed lines) and in PB electrolyte with added scavenger (solid lines), under AM 1.5G illumination; (**d**) PEC performances of P-BiVO_4_ (black line) and P-BiVO_4_/TiO_2_ (sky-blue line) photoanodes in general potassium borate (PB) electrolyte (dashed lines) and in PB electrolyte with added scavenger (solid lines). (**e**) Schematic for charge kinetics at the bulk, surface, and interface of the BiVO_4_ with optimized overlayer. Reprinted with permission from Ref. [163], Copyright © 2025, Wiley-VCH GmbH.

Lei et al. demonstrated that conformal NiO_x_ overlayers deposited via ALD, combined with the deliberate addition of NaVO_3_ to the electrolyte, provided a synergistic stabilization effect. The NiO_x_ coating suppressed surface charge recombination and facilitated hole transfer by stabilizing Bi–O bonds, while dissolved vanadate species helped re-establish chemical equilibrium and compensate for V loss. This dual-protection strategy enabled NiO_x_/BiVO_4_ photoanodes to achieve an applied-bias photon-to-current efficiency (ABPE) of 2.05% with a fill factor of 47.1%, and, remarkably, operational durability exceeding 2100 h under continuous PEC operation. The long-term stability and proposed anticorrosion mechanism of the NiO_x_-protected BiVO_4_ electrodes are illustrated in Figure 15a,b [162].

More recently, Lee et al. introduced a complementary approach by selectively engineering surface oxygen vacancies (VO) via a surface chemical reduction (SCR) process, followed by deposition of an ultrathin TiO_2_ layer (≈5 nm) via ALD. The VO-rich surface promoted strong oxygen-end networking with TiO_2_, yielding a nearly pinhole-free protective layer that minimized interfacial recombination while maintaining efficient charge tunneling. When further coupled with a cobalt phosphate (CoPi) oxygen evolution catalyst, the optimized SCR-BiVO_4_/TiO_2_/CoPi photoanode exhibited a stable photocurrent density of 3.9 mA/cm^2^ at 1.23 *V_RHE_*, with charge transfer efficiency of up to 97% and sustained photostability for over 50 h while generating stoichiometric *H*_2_ and *O*_2_. The PEC performances in different electrolytes and the schematic of charge kinetics in this optimized multilayer system are shown in Figure 15c–e. [163].

These findings confirm that electrolyte composition is a critical factor in BiVO_4_ stability. By adding protective species (e.g., phosphate buffers, vanadium ions) or coupling with passivation layers (e.g., NiO_x_), the dissolution rate can be significantly suppressed while preserving efficient hole transfer, making electrolyte engineering a powerful tool for enabling durable PEC water splitting devices.

#### 4.8.3. Synergistic Surface–Electrolyte Protection Strategies

**Figure 16:**
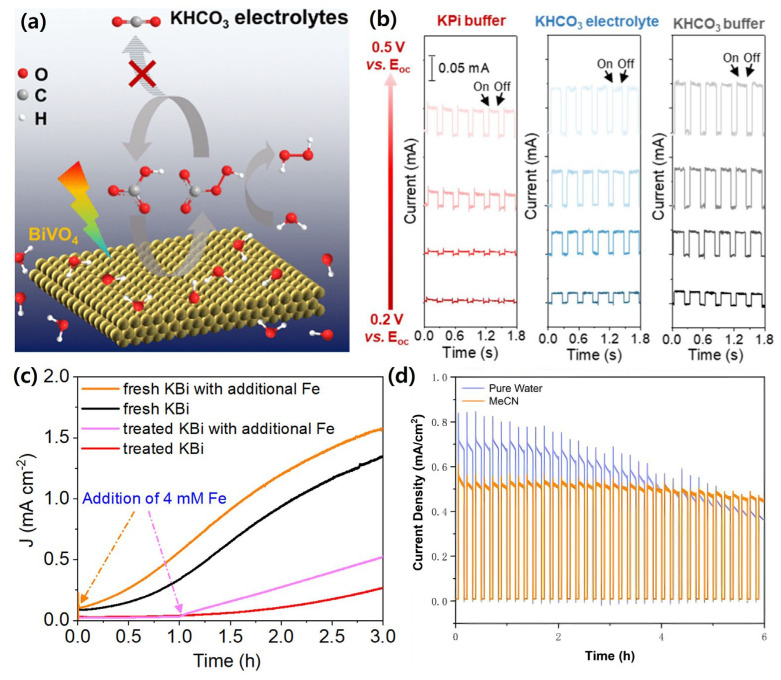
(**a**) Schematic illustration of water splitting cell configuration in KHCO_3_ electrolyte. Reprinted with permission from Ref. [164], Copyright © 2025, American Chemical Society. (**b**) Transient photocurrent response of photoanodes in KPi buffer, unbuffered KHCO_3_, and CO_2_-saturated KHCO_3_ electrolyte under chopped illumination. Reprinted with permission from Ref. [164], Copyright © 2025, American Chemical Society. (**c**) *J*–*t* curves of BiVO_4_ photoanodes at 0.6 *V_RHE_* before and after Fe impurity removal, with photocurrent change upon Fe^2+^ addition. Reprinted with permission from Ref. [165], Copyright © 2024, Wiley-VCH GmbH. (**d**) Stability of BVO photoanodes in aqueous and MeCN buffers containing 0.5 M NaHCO_3_ (pH 9) over 6 h. Reprinted with permission from Ref. [166], Copyright © 2024, MDPI.

The identity of the electrolyte—including buffer anions/cations and pH—has been reported to govern not only interfacial oxygen evolution kinetics but also the chemical pathways of photocorrosion in BiVO_4_. In phosphate electrolytes, competitive reactions between water oxidation and lattice dissolution proceed via phosphate–bismuth surface interactions, whose rates accelerate under illumination and elevated potentials, as illustrated in Figure 16a. These findings emphasize that stability should be assessed alongside kinetics rather than inferred from photocurrent alone, as demonstrated by the contrasting stability profiles in Figure 16b [164].

In borate buffers, a frequently observed “activation” effect has been attributed to trace Fe impurities, which deposit as an ultrathin oxyhydroxide layer that passivates surface traps and lowers interfacial resistance. Under optimized conditions, bare BiVO_4_ photoanodes have been reported to reach ≈4.5 mA/cm^2^ at 1.23 *V_RHE_* and surface Fe activation layers further boost photocurrent and enhance photostability by promoting Fe-layer formation on the surface. Comparable enhancements across Li^+^ Na^+^, and K^+^ borates support the view that Fe-mediated passivation, rather than alkali identity, governs this activation behavior. The photocurrent response before and after Fe impurity removal, as well as the effect of Fe^2+^ addition, are presented in Figure 16c [165]. Nevertheless, quantitative correlations between Fe concentration and performance gain remain limited and merit systematic study.

Beyond conventional phosphate and borate systems, bicarbonate electrolytes—including mixed or non-aqueous variants—have been shown to mitigate vanadium leaching, especially when supplemented with saturated V^5+^. This approach reduces morphological degradation and preserves optical density over extended operation, underscoring electrolyte engineering as a viable lever for durability improvement. The long-term stability of BiVO_4_ photoanodes in aqueous and mixed MeCN/NaHCO_3_ electrolytes is shown in Figure 16d [166].

Electrochemical conditioning protocols, such as controlled pre-bias or photocharging sequences, have also been demonstrated to enhance operational stability by tuning surface states and optimizing interfacial charge transfer pathways. These effects are synergistic with electrolyte composition, suggesting that combined strategies may yield more durable BiVO_4_ photoanodes [167].

In summary, electrolyte design directly shapes both surface kinetics and corrosion chemistry in BiVO_4_ photoanodes. Phosphate media can accelerate degradation without stabilization measures [164]; borate buffers “activate” BiVO_4_ via Fe-assisted passivation [165]; bicarbonates, particularly with V^5+^ supplementation, suppress vanadium loss and slow morphological decay [166]; and electrochemical conditioning provides an orthogonal route to stabilize interfacial dynamics [167]. Future studies are encouraged to report kinetic and stability metrics in parallel, clearly specify buffer composition and pH ranges, and combine electrolyte engineering (e.g., Fe-controlled borate or V^5+^-supplemented bicarbonate) with conditioning protocols to enable reproducible and robust long-term PEC operation.

## 5. Emerging Applications of BiVO_4_ Photoelectrodes in Solar Water Splitting and Beyond

While materials strategies (doping, heterojunctions, HTLs/overlayers, and electrolyte engineering) have substantially improved BiVO_4_ at the electrode level, practical relevance is established only in overall water splitting devices, where a BiVO_4_ photoanode is paired with a photocathode or photovoltaic element to operate unassisted (0 V external bias) or at low bias. This section surveys recent device architectures (PEC–PEC tandems, PEC–PV hybrids, monolithic vs. wired configurations) and applies a consistent set of performance benchmarks under AM 1.5G: (i) *J_PEC_* at 1.23 *V_RHE_* to normalize anodic *OER* kinetics, (ii) STH (%) as a device-level energy conversion metric, (iii) electrolyte/pH and applied bias for fair comparison, and (iv) durability (h) with supporting evidence (e.g., gas-tight collection and faradaic efficiency) when available. Building on these data, we discuss such as energy-level matching in tandems, reduction in ohmic/contact losses, membrane and electrode spacing design with hydrodynamic considerations, as well as pre-conditioning protocols and corrosion-mitigating electrolytes (e.g., impurity-controlled borate or vanadate-buffered media). The accompanying comparison table includes only reports with complete entries to provide a quantitative basis for assessing genuine progress and guiding the integration of BiVO_4_ into scalable modules.

Table 5 summarizes BiVO_4_-based photoanodes for overall water splitting under unbiased conditions, highlighting that the integration of oxygen evolution catalysts, Mo doping, surface nano-structuring, and organic hole transport layers can substantially enhance both *J_PEC_* and STH efficiencies. Among the reported systems, Mo:BiVO_4_ combined with a polycarbazole HTL and NiFeCoO_x_ OEC exhibits one of the highest performances (*J_PEC_* ≈ 6.6 mA/cm^2^, STH ≈ 9%), emphasizing the importance of optimizing charge separation and catalytic activity in achieving high-efficiency photoanodes. [110].

### 5.1. BiVO_4_-Based Tandem System for Comprehensive Water Splitting

Achieving efficient overall water splitting without external bias requires that the top photoanode generates sufficient photovoltage while maintaining strong light absorption and operational stability. BiVO_4_, with a bandgap of ~2.4 eV and robust visible light absorption, remains a promising candidate. To overcome its intrinsic limitation in photovoltage, researchers have explored tandem PEC configurations that combine BiVO_4_ with additional light-harvesting materials.

**Figure 17:**
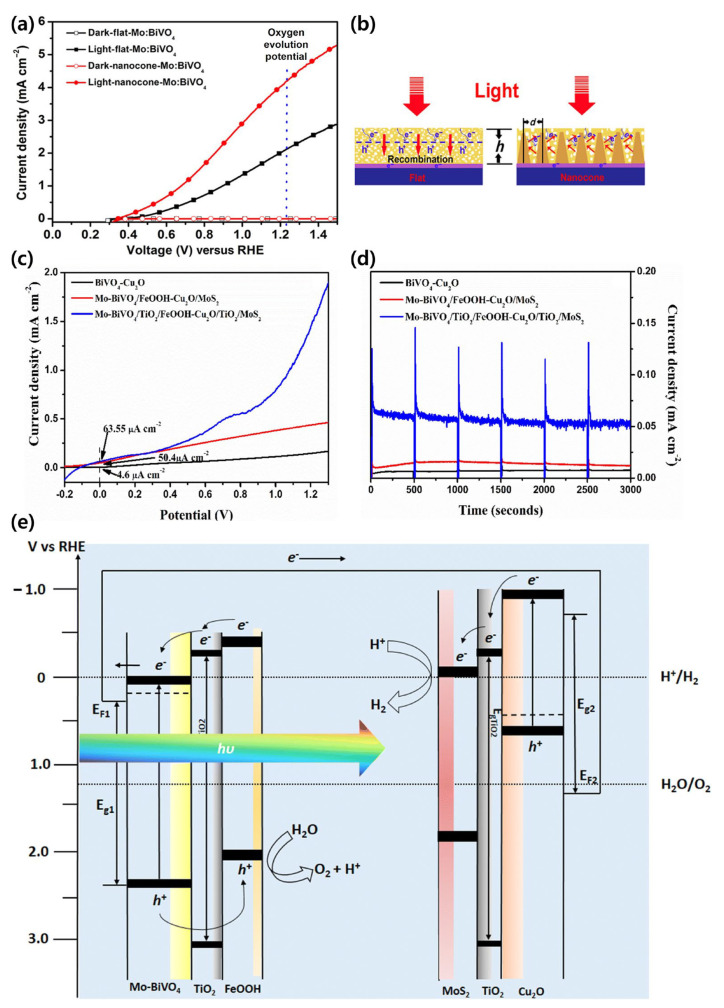
(**a**) Schematic illustration of the optical absorption mechanism and electron transport of nanoporous BiVO_4_ on the flat substrate and the conductive nanocone substrate. (**b**) *J–V* curves of the Mo:BiVO_4_ on the FTO-coated glass and the nanocone substrate tested in a 0.5 M KH_2_PO_4_ buffer solution (pH 7). Reprinted with permission from Ref. [33], Copyright © 2016, American Association for the Advancement of Science. (**c**) 2-electrode LSV response of BiVO_4_-Cu_2_O, Mo-BiVO_4_/FeOOH-Cu_2_O/MoS_2_ and Mo-BiVO_4_/TiO_2_/FeOOH-Cu_2_O/TiO_2_/MoS_2_ tandem cells and (**d**) unassisted stability test (*j–t*); (**e**) energy band diagram of BiVO_4_–Cu_2_O tandem PEC cell with respect to RHE potential. Reprinted with permission from Ref. [53], Copyright © 2022, Royal Society of Chemistry.

A notable example is a BiVO_4_ nanocone/Fe(Ni)OOH photoanode coupled with a perovskite solar cell. Under AM 1.5G illumination, this system achieved unassisted overall water splitting with a STH efficiency of up to 6.2%, leveraging complementary light absorption and efficient interfacial charge transfer. The standalone PEC BiVO_4_-based component exhibited a photocurrent density of 5.82 ± 0.36 mA/cm^2^ at 1.23 *V_RHE_* The optical absorption mechanism of the nanocone substrate and the corresponding *J*–*V* curves confirming this photocurrent enhancement are shown in Figure 17a,b [33].

Another recent advancement involves a monolithically integrated tandem system combining a BiVO_4_ photoanode with a Cu_2_O photocathode, both protected by conformal layers. Sitaaraman et al. fabricated a Mo-BiVO_4_/TiO_2_/FeOOH photoanode paired with a Cu_2_O/TiO_2_/MoS_2_ photocathode, forming an unassisted tandem PEC cell. The TiO_2_ layers serve as protective barriers and facilitate charge extraction, whereas FeOOH and MoS_2_ act as cocatalysts to accelerate the *OER* and HER. Under AM 1.5G illumination, the individual electrodes delivered ~0.81 mA/cm^2^ (photoanode) and –1.88 mA/cm^2^ (photocathode), and the assembled tandem achieved an unassisted current density of approximately 65.3 µA/cm^2^, with enhanced stability compared to unprotected devices. The LSV response, unassisted stability test, and energy band diagram of these tandem PEC devices are illustrated in Figure 17c–e [53].

The solar-to-hydrogen efficiency for tandem devices is calculated as

(9) $$\eta_{STH}\left(\%\right)={\left[\frac{J_{sc}\left(\frac{mA}{{cm}^{2}}\right)\times \left(1.23V\right)\times \eta_{F}}{P_{Total}\left(\frac{mW}{{cm}^{2}}\right)}\right]}_{AM1.5G}$$

Here, *J_sc_* represents the short-circuit photocurrent density corresponding to the rate of hydrogen generation expressed as current density, *η_F_* is the Faradaic efficiency for hydrogen production (typically near 100%), and *P_Total_* denotes the total solar irradiance intensity, set at 100 mW/cm^2^ under AM 1.5G conditions. By overlaying the current–voltage (*J*–*V*) curve of the photoanode with those of the photocathode or photovoltaic cells, the operating point of the tandem device can be determined, defined by the short-circuit photocurrent density at 0 V applied bias. However, the actual device performance often falls short of the predicted operating point due to overpotentials between the two electrodes. This discrepancy arises because the *J*–*V* curves of individual half-cells (including photovoltaics), referenced to the reversible hydrogen electrode (RHE) scale, do not account for resistive losses such as ohmic resistance and pH gradients present between the components.

### 5.2. PEC Cells for the Generation of Value-Added Chemicals

While PEC systems are primarily designed for overall water splitting, there is increasing interest in their application for the selective synthesis of value-added chemicals, such as hydrogen peroxide (*H*_2_*O*_2_), via partial oxidation or reduction under solar illumination. *H*_2_*O*_2_ can be selectively produced when the PEC system is appropriately engineered, exploiting its redox potentials of 1.77 *V_RHE_* (via 2*e*^−^ water oxidation) and 0.68 *V_RHE_* (via oxygen reduction).

The fundamental half-reactions for PEC-based *H*_2_*O*_2_ generation are summarized as follows:

(10) $$\mathrm{Oxygen}\;\mathrm{evolution}\;\mathrm{reaction}:2H_{2}O+4h^{+}\to 4H^{+}+O_{2},\;E^{{}^{\circ}}=1.23{\;V}_{\mathrm{RHE}}$$

(11) $$H_{2}O_{2}\mathrm{production}\;\mathrm{by}\;\mathrm{water}\;\mathrm{oxidation}:2H_{2}O+2h^{+}\to 2H^{+}+H_{2}O_{2},\;{\;E}^{{}^{\circ}}=1.77\;V_{\mathrm{RHE}}$$

(12) $$\mathrm{Hydrogen}\;\mathrm{evolution}\;\mathrm{reaction}:2H^{+}+2e^{-}\to H_{2},\;E^{{}^{\circ}}=0.0{\;V}_{\mathrm{RHE}}$$

(13) $$H_{2}O_{2}\mathrm{production}\;\mathrm{by}\;\mathrm{oxygen}\;\mathrm{reduction}:O_{2}+2H^{+}+2e^{-}\to H_{2}O_{2},\;E^{{}^{\circ}}=0.68{\;V}_{\mathrm{RHE}}$$

These reactions highlight that by controlling the PEC environment and electrode materials, the pathways for partial oxidation or reduction can be preferentially promoted, enabling efficient and selective *H*_2_*O*_2_ production.

**Figure 18:**
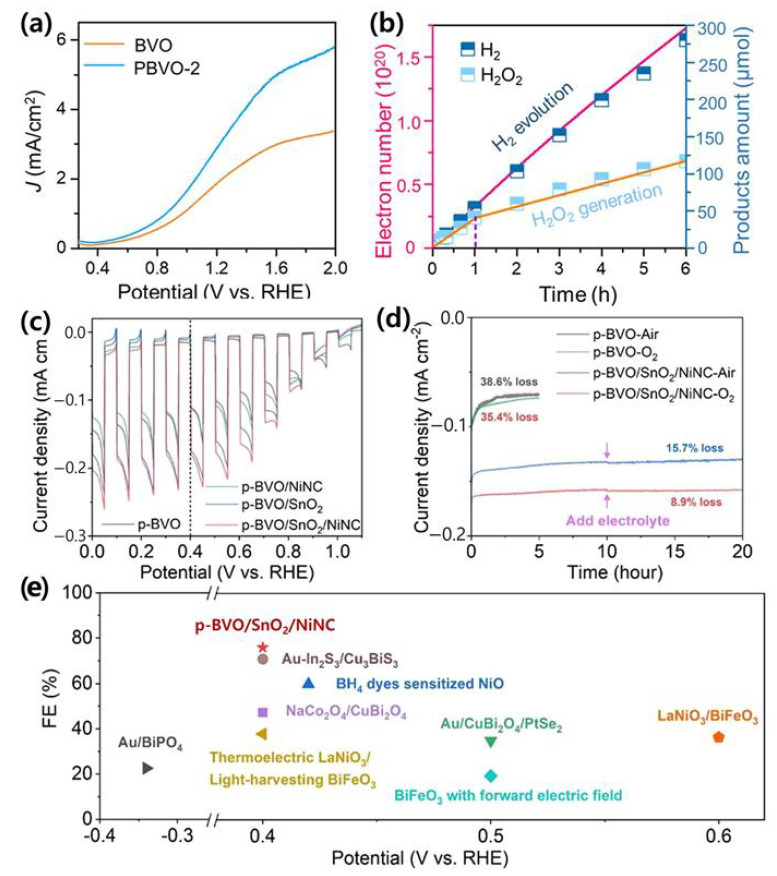
(**a**) LSV curves of BVO and PBVO-2 photoanodes in 1 M NaHCO_3_ electrolyte under AM 1.5G illumination. (**b**) Plots of the theoretical charge number obtained from the *J*–*t* curves and the actual quantities of *H*_2_ and *H*_2_*O*_2_ Reprinted with permission from Ref. [173], Copyright © 2023, American Chemical Society. (**c**) Chopped *J*–*V* curves, (**d**) *I*–*t* curves under air and oxygen conditions of photoanodes. (**e**) Comparison of Faradaic efficiency of *H*_2_*O*_2_ generation by different photocathodes. Reprinted with permission from Ref. [174], Copyright © 2024, American Chemical Society.

Recent advances have highlighted BiVO_4_-based systems as promising platforms for PEC hydrogen peroxide production. Wan et al. demonstrated that phosphate-modified BiVO_4_ (PBVO) photoanodes undergo dynamic anion exchange with bicarbonate (HCO_3_^−^) at the semiconductor–electrolyte interface, which accelerates charge transfer and enhances the selectivity of the two-electron water oxidation pathway. This modification achieved an average Faradaic efficiency of 82.6% (maximum 92.1%) with a *H*_2_*O*_2_ production rate of 66.5 µmol/h, while the formation of H_2_PO_4_^−^ intermediates suppressed *H*_2_*O*_2_ overoxidation. The LSV curves and charge balance plots confirming the selective *H*_2_*O*_2_ production on PBVO-2 photoanodes are shown in Figure 18a,b [173]. Under AM 1.5G illumination in a H-type PEC cell, PBVO-2 photoanodes exhibited ~1.6-fold higher photocurrent density than pristine BiVO_4_, maintained a solar-to-hydrogen efficiency of 1.27%, and enabled continuous *H*_2_*O*_2_ accumulation up to 2.34 × 10^−3^ M.

Complementarily, Shi et al. reported a heterojunction photocathode based on p-BiVO_4_/SnO_2_/NiNC, which delivered a *H*_2_*O*_2_ generation rate of 65.46 µmol/h with a Faradaic efficiency of 76.1%. LSV revealed significantly enhanced photocurrent density at 0.4 *V_RHE_* compared to pristine p-BVO and other oxide-modified photocathodes (TiO_x_, NiO_x_, ZnO), confirming the charge transport benefit of the SnO_2_ interlayer. The IPCE reached a maximum of 8.5%, and *I*–*t* measurements demonstrated excellent stability with photocurrent maintained above 0.15 mA/cm^2^ and only 8.9% loss after 20 h of operation under *O*_2_-saturated conditions. Integration with a Mo-doped BiVO_4_ photoanode in a tandem cell achieved nearly quantitative *H*_2_*O*_2_ selectivity. The chopped *J*–*V* curves, transient current responses, and Faradaic efficiency data supporting this high selectivity are presented in Figure 18c–e [174].

Collectively, these studies demonstrate that surface anion modulation and rational heterojunction design enable efficient and selective PEC conversion of water to *H*_2_*O*_2_, establishing BiVO_4_-based systems as a viable platform for solar-driven production of value-added oxidants.

## 6. Challenges and Perspectives

Despite significant advances in BiVO_4_ electrodes over the past four decades, these materials still fall short of the requirements for practical applications, highlighting several challenges and opportunities.

Photocurrent Density Limitations: While recent reports have achieved photocurrent densities up to 6.0 mA/cm^2^ at 1.23 *V_RHE_*, this performance remains below the theoretical limit of 7.5 mA/cm^2^. Improving PEC water splitting efficiency, therefore, requires enhancements in light absorption, charge transfer, and carrier separation. A deeper understanding of factors influencing these processes could guide the design of more efficient BiVO_4_ electrodes. In particular, the role of crystal orientation on PEC performance remains underexplored, representing a promising avenue for future research. Additionally, synthesizing BiVO_4_ electrodes with multicomponent and multifunctional catalysts could provide additional active sites for hydrogen and oxygen evolution while promoting more effective charge separation.

Challenges in Understanding PEC Mechanisms: Understanding the fundamental PEC reaction mechanisms remains challenging. Most studies have prioritized performance improvements, often at the expense of mechanistic insight. Limited access to advance in situ techniques has hindered in-depth exploration of water oxidation on BiVO_4_. Approaches such as infrared spectroscopy, X-ray spectroscopy, and transient absorption spectroscopy could enable observation of reaction dynamics and intermediates at the molecular or atomic scale. Developing these methodologies will be essential for advancing the mechanistic understanding of PEC water oxidation.

Scaling Up BiVO_4_-Based Photoanodes: Current research largely involves small-area BiVO_4_ photoanodes (~1.0 cm^2^), whereas practical deployment demands large-area materials with high uniformity and crystallinity. Fabrication via electroplated BiOI conversion faces challenges in maintaining uniformity across large substrates due to increased resistance, nonuniform films, and nonlinear diffusion of reactants and products. Developing scalable and cost-effective synthesis strategies for large-area, high-quality photoelectrodes will be critical for commercial applications.

Design of Tandem PEC Systems: Tandem configurations using dual- or three-electrode setups are common in practical PEC systems. A feasible approach for real-world devices involves compartmentalization with membranes, along with careful optimization of electrode spacing, membrane design, and pressure balance. Tandem PEC modules often integrate multijunction or perovskite photovoltaic cells, requiring precise matching of energy levels between the photoanode and photocathode. Further engineering efforts, including fluid dynamics optimization, are needed to maximize device performance under operational conditions.

Advanced/Operando Spectroscopy and Diagnostics: To convert performance gains into durable operation, buried-interface processes must be observed under realistic bias, illumination, and electrolyte conditions. High-energy-resolution X-ray absorption (HERFD-XANES/XAS) at the Bi L and V K edges can track oxidation-state shifts, ligand fields, and local coordination during *OER*, revealing whether interlayers/overlayers stabilize Bi–O and V–O polyhedra or merely delay dissolution. Ambient-pressure/operando XPS resolves surface terminations, adsorbates (e.g., phosphate/borate/vanadate), and HTL-derived functional groups that modulate band bending and recombination velocities. Operando GIWAXS/XRD identifies phase transformations (e.g., BiPO_4_ signatures, amorphization), while Raman/IR spectroelectrochemistry reports reaction intermediates and lattice strain. Charge-dynamics probes—IMPS/EIS to deconvolute η_sep vs. η_trans, transient absorption (fs–µs) and time-resolved photoluminescence to quantify interfacial transfer constants, and UV–Vis spectroelectrochemistry to monitor polaron/charge accumulation—link kinetic rate constants to optical populations. Finally, pairing photocurrent with operando dissolution (e.g., ICP-MS in a scanning flow cell or EQCM) closes the gap between apparent activity and true stability by quantifying Bi/V loss in real time.

Standardization and Reporting of Stability: To ensure comparability across studies, we recommend (i) co-reporting *J*–*V* (or ABPE) with operando dissolution traces on the same electrode; (ii) specifying light intensity (AM 1.5G), temperature, pH, buffer composition and impurities (e.g., trace Fe), bias protocols (hold vs. scan; scan rate), and operation time; (iii) adopting common endurance metrics (e.g., current retention and dissolution yield after 10–24 h at a defined potential); and (iv) providing raw spectra and analysis scripts for XAS/XPS/Raman together with IMPS/EIS fitting models. Such practices reduce ambiguity between kinetic- and corrosion-driven losses and accelerate translation to device design.

Diagnostics for Scale-Up: As devices move beyond ~1 cm^2^, spatial non-uniformities dominate. Integrating spatially resolved tools—scanning photoelectrochemical microscopy (SPECM), light-beam-induced current mapping, micro-XRD/µ-XRF, or X-ray/optical tomography—can identify local recombination hot spots, catalyst delamination, and electrolyte transport limitations, informing fluid dynamics optimization and contact/grid design for large-area modules.

Given these multifaceted challenges, interdisciplinary collaboration is increasingly important. Combining expertise from materials science, physical chemistry, surface science, and advanced characterization techniques will be essential to develop more stable and efficient BiVO_4_-based photoelectrodes for PEC water splitting.

## Figures and Tables

**Table 1:** Summary of materials, fabrication method, electrolyte condition, and PEC performance of BiVO_4_-based photoanodes.

Materials	Fabrication Method	Annealing Condition (Temp., Time, Atmosphere)	Electrolyte Condition	Photocurrent Density @ 1.23 *V_RHE_* (mA/cm^2^)
Bare BiVO_4_ [47]	Electrodeposition BiOI + VO(acac)_2_ drop	450 °C, 2 h, air	0.1 M Na_2_SO_4_ (pH 6)	0.65
NiOOH/FeOOH/BiVO_4_ [48]	Electrodeposition BiOI + VO(acac)_2_ drop + cocatalysts deposition	450 °C, 2 h, air	0.1 M Na_2_SO_4_	~1.3
CdS/NiFe-LDH/BiVO_4_ [49]	BiOI + VO(acac)_2_ + CdS hydrothermal + NiFe-LDH deposition	450 °C, 2 h, air	0.5 M Na_2_SO_4_	3.10
BiVO_4_/NiO composite [50]	BiOI + VO(acac)_2_ conversion + NiO HT	450 °C, 2 h, air	0.5 M Na_2_SO_4_	1.20
CoV-LDH/Ag/BiVO_4_ [51]	BiOI + VO(acac)_2_→CoV-LDH/Ag coating	450 °C, 2 h, air	0.5 M Na_2_SO_4_ + 0.1 M glycerol	7.15
Bare BiVO_4_ [52]	BiOI electrodeposition + V_2_O_5_ electrodeposition + calcination	475 °C, 2 h, air	0.5 M potassium borate (pH 9.5)	2.2
Mo-BiVO_4_/TiO_2_/FeOOH [53]	BiOI + VO/(Mo dopant) + TiO_2_ + FeOOH	450 °C, 2 h, air	0.1 M Na_2_SO_4_	0.81
BiVO_4_/PbS QDs/ZnS [54]	BiOI + VO(acac)_2_→BiVO_4_ + PbS/ZnS (SILAR)	450 °C, 2 h, air	0.5 M KH_2_PO_4_ + 1.0 M Na_2_SO_3_	5.19

**Table 2:** Summary of modification strategies for enhancing the PEC performance of BiVO_4_-based photoanodes.

Techniques	Materials/Structures/Methods	Functionality
1. Synthesis and Film Morphology Engineering	Electrodeposition [84] Hydrothermal synthesis [85] Hollow nanospheres [86] Metal–organic decomposition [86] RF sputtering [87]	(1) Controls morphology and film thickness. (2) Increases surface area. (3) Improves light absorption and bulk charge transport (*η_bulk_*). (4) Precisely controls composition and film density. (5) Enables conformal coating on complex nanostructures.
2. Doping	Mo, W [88]. Mo and W [89]. Ti, Zr [90]	(1) Increases bulk conductivity. (2) Tunes the bandgap. (3) Reduces bulk recombination.
3. Surface and Cocatalyst Modification	FeOOH, NiOOH [20] NiOOH thin layer [91] RuO_2_, IrO_2_ [92] CoPi [93]	(1) Lowers *OER* overpotential. (2) Improves surface reaction kinetics (*η_surf_*). (3) Increases chemical stability. (4) Accelerates oxygen evolution reaction. (5) Enhances charge transfer efficiency at the surface.
4. Heterojunction Engineering	WO_3_ [94] Cu_2_O [95] BiVO_4_/MoS_2_ [96] BiVO_4_/CdS/TiO_2_ [97] BiVO_4_/Ag NPs [98] PbS Quantum Dots [54] NiO_x_ [99]	(1) Increases electron–hole separation efficiency. (2) Improves quantum efficiency. (3) Enhances light absorption. (4) Boosts performance via surface resonance effect. (5) Provides a direct pathway for hole extraction. (6) Expands absorption into the near-infrared and enhances energy conversion efficiency.
5. Post-treatment and Passivation	Plasma (Ar, O_2_) [100] Controlled annealing [101] Strain engineering [102] Small organic compounds [101,102] ALD Al_2_O_3_/TiO_2_ [103]	(1) Passivates surface defects. (2) Improves crystallinity. (3) Tunes electronic band structure. (4) Increases stability and reduces recombination. (5) Creates a protective layer against photocorrosion.
6. Other Treatment Methods	Antireflection coatings [98] Controlled defect creation [104] Laser-sintering [105]	(1) Enhances photon absorption. (2) Improves electrical conductivity. (3) Promotes desired defect states for enhanced activity.

**Table 3:** Summary of representative cocatalyst and surface overlayer strategies for enhancing the PEC performance of BiVO_4_ photoanodes.

Structure/Strategy	Cocatalyst or Overlayer	Performance Highlight	Key Effect	Ref.
One-step PEC deposition	FeOOH (inner) + Co–Sil (outer)	6.10 mA/cm^2^ @1.23 V	Dual-layer cocatalyst boosts charge separation and reduces recombination	[115]
Fluoride-assisted in situ passivation	F^−^ ions	Long-term stability >100 h @0.6 V	Surface passivation and cocatalyst reactivation	[116]
Porphyrin-based surface ligand	Co–He (Co–O–V linkage)	5.3 mA/cm^2^ @1.23 V, V_on_ = 0.07 V	Low overpotential and efficient hole transfer	[117]
Organic ligand modification	Co^2+^ + BTC ligand	4.82 mA/cm^2^, onset 0.22 V	Surface passivation + cocatalyst anchoring	[118]
Magnetic overlayer	Co-doped Fe_3_O_4_	1.9× higher *OER* activity, Faradaic efficiency >85%	Improves surface kinetics and protects BiVO_4_	[119]
Dual cocatalyst immersion	FeOOH + Co(OH)_2_	2.56 mA/cm^2^, 71.6% retention (10 h)	Synergistic catalytic enhancement + stability	[120]
Bilayer MOF cocatalyst	Fe-MOF/Ni-MOF	1.80 mA/cm^2^, V_on dropped from 0.9 V to 0.69 V	Facilitated interfacial charge transfer	[121]
Room-temp photodeposition	Co-Pi, Ni-Bi, Mn-Pi/BiVO_4_	Hole transfer efficiency up to 94.5 % @1.23 *V_RHE_*	Conformal, uniform cocatalyst deposition	[122]
Facet-selective cocatalyst loading	Selective facet-modified MnO_x_	0.74 mA/cm^2^ @1.23 *V_RHE_*	Maximize catalytic activity by crystal facet control	[123]
In situ solvothermal growth	COF–Azo	1.38 mA/cm^2^ @1.23 *V_RHE_*	Improves carrier separation, lowers impedance, and accelerates *OER*	[124]
Bulk Mo doping + surface molecular catalyst deposition	CoPOM	4.32 mA/cm^2^ @1.23 *V_RHE_*	Conductivity enhancement + catalytic activation	[125]

**Table 4:** Summary of *η_sep_* and PEC activity in modified BiVO_4_ photoanodes. *η_sep_* and *η_inj_* extracted under AM 1.5G; all values measured at 1.23 *V_RHE_* unless noted.

Photoanodes	*J_PEC_* @1.23 *V_RHE_* (mA/cm^2^)	*η_sep_* (%)	*η_inj_* (%)	Modification Method	Ref.
WO_3_/S:Bi_2_O_3_/(Ga,W):BiVO_4_/Co-Pi	5.10	N/R	N/R	Interface design	[140]
Co_3_O_4_/BiVO_4_	~2.3	N/R	N/R	Cocatalyst interface	[141]
Plasma-treated N-doped BiVO_4_	1.39	~4.6× higher vs. pristine	N/R	N doping + oxygen vacancies	[142]
Co:BiVO_4_/Mo:BiVO_4_	2.09	77.8	86.5	Homojunction (doped layers)	[139]
Zn:BiVO_4_/Mo:BiVO_4_	2.70	65.0	89.0	Homojunction	[143]
Ni-BiVO_4_/FeOOH	3.02	N/R	73.3	Homojunction (+*OER* overlayer)	[144]
BiVO_4_/SnO_2_ (heterostructure)	5.61	97	N/R	Heterojunction + cocatalyst	[145]
O_v_-BiVO_4_ (VO_x_ engineered)	6.29	94	96	VO_x_ (oxygen vacancy-engineered)	[146]

**Table 5:** Comparison of BiVO_4_ photoanodes for overall water splitting with complete reporting.

Photoanodes	Bias	Electrolyte	*J_PEC_* @1.23 *V_RHE_* (mA/cm^2^)	STH (%)	Ref
NiOOH/FeOOH/BiVO_4_/SnO_2_//TTO//TOPCon-Si	Unbiased	1.0 M potassium borate, pH 9	1.40	1.72	[168]
Nanocone/Mo:BiVO_4_/Fe(Ni)OOH	Unassisted	Phosphate buffer, pH 7	5.82 ± 0.36	6.2	[33]
BiVO_4_/NiOOH/FeOOH (top)//Cu_2_O/CuO/TiO_2_ (bottom)	Unassisted	0.1 M Na_2_SO_4_, pH 6	2.05	0.27	[169]
BiVO_4_/FeOOH (oxygen vacancy gradient; FeOOH OEC)	Unassisted	1 M borate buffer (pH ≈ 9)	7.0	8.4	[170]
BiVO_4_/Cu_2_O/NiFe-LDH	Unassisted	0.1 M Na_2_SO_4_, pH 6	5.01	1.18	[171]
Mo:BiVO_4_ + polycarbazole HTL (CPF-TCB) + NiFeCoO_x_ OEC	Unassisted	K–borate buffer (pH ≈ 9–10)	≈6.6	~9	[110]
CoPi/W:BiVO_4_/Ni	Unassisted	K-phosphate buffer (pH 7)	1.5	2.1–6.3	[172]

## Data Availability

Not applicable.
